# Deubiquitinating enzyme USP39 promotes the growth and metastasis of gastric cancer cells by modulating the degradation of RNA-binding protein RBM39

**DOI:** 10.1016/j.jbc.2024.107751

**Published:** 2024-09-10

**Authors:** Chengpiao Lu, Yunxin Cai, Shenglong Wu, Yuhong Wang, Jia-Bin Li, Guoqiang Xu, Jingjing Ma

**Affiliations:** 1 Jiangsu Key Laboratory of Neuropsychiatric Diseases and College of Pharmaceutical Sciences, Jiangsu Province Engineering Research Center of Precision Diagnostics and Therapeutics Development, Jiangsu Key Laboratory of Preventive and Translational Medicine for Geriatric Diseases, Suzhou Key Laboratory of Drug Research for Prevention and Treatment of Hyperlipidemic Diseases, Soochow University, Suzhou, Jiangsu, China; 2 Department of Pathology, The First Affiliated Hospital of Soochow University, Suzhou, Jiangsu, China; 3 Suzhou International Joint Laboratory for Diagnosis and Treatment of Brain Diseases, College of Pharmaceutical Sciences, Soochow University, Suzhou, Jiangsu, China; 4 MOE Key Laboratory of Geriatric Diseases and Immunology, Suzhou Medical College of Soochow University, Suzhou, Jiangsu Province, China; 5 Department of Pharmacy, The Fourth Affiliated Hospital of Soochow University, Suzhou Dushu Lake Hospital, Medical Center of Soochow University, Suzhou, Jiangsu, China

**Keywords:** RBM39, USP39, gastric cancer, growth, colony formation, migration, invasion, ubiquitination

## Abstract

It has been revealed recently that the RNA-binding motif protein RBM39 is highly expressed in several cancers, which results in poor patient survival. However, how RBM39 is regulated in gastric cancer cells is unknown. Here, affinity purification-mass spectrometry and a biochemical screening are employed to identify the RBM39-interacting proteins and the deubiquitinating enzymes that regulate the RBM39 protein level. Integration of the data obtained from these two approaches uncovers USP39 as the potential deubiquitinating enzyme that regulates RBM39 stability. Bioinformatic analysis discloses that USP39 is increased in gastric cancer tissues and its elevation shortens the duration of overall survival for gastric cancer patients. Biochemical experiments verify that USP39 and RBM39 interact with each other and highly colocalize in the nucleus. Expression of USP39 elevates while *USP39* knockdown attenuates the RBM39 protein level and their interaction regulates this modulation and their colocalization. Mechanistic studies reveal that USP39 reduces the K48-linked polyubiquitin chains on RBM39, thus enhancing its stability and increasing the protein level by preventing its proteasomal degradation. USP39 overexpression promotes while its knockdown attenuates the growth, colony formation, migration, and invasion of gastric cancer cells. Interestingly, overexpression of RBM39 partially restores the impact of USP39 depletion, while *RBM39* knockdown partially abolishes the effect of USP39 overexpression on the growth, colony formation, migration, and invasion of gastric cancer cells. Collectively, this work identifies the first DUB for RBM39 and elucidates the regulatory functions and the underlying mechanism, providing a possible alternative approach to suppressing RBM39 by inhibiting USP39 in cancer therapy.

Gastric cancer ranks among the most common cancers globally, characterized by high incidence and mortality (1). Although gastric cancer can be treated with surgery, chemotherapy, radiotherapy, targeted therapy, and immunotherapy (2), the 5-year survival rate is still low for patients with advanced gastric cancer because of drug resistance and metastasis (3). Therefore, there is an unprecedented need to discover new molecules that regulate the progression of gastric cancer. Recently it has been discovered that the RNA-binding motif protein RBM39 is highly expressed in tissues from several cancer patients and its high expression is associated with reduced patient survival (4). The ubiquitination and proteasomal degradation of RBM39 could be induced by sulfonamide compounds such as indisulam, E7802, tasisulam, and chloroquinoxaline sulfonamide, thereby inhibiting cancer cell growth (5). The downstream targets of RBM39 were explored and the differential gene expression and alternative splicing it regulates were identified by RNA-seq (6, 7). However, the upstream regulators for RBM39 are not systematically studied.

Ubiquitination is a process that covalently conjugates ubiquitin to specific residues of protein substrates through a series of reactions catalyzed by ubiquitin-activating enzymes E1, ubiquitin-conjugating enzymes E2, and ubiquitin ligases E3 (8). This process can also be inverted by deubiquitinating enzymes (DUBs), thereby maintaining the balance between the modified form and the nonmodified form and modulating the associated functions (9). The types of ubiquitination determine the fate of the modified substrates. For example, K48-linked polyubiquitin chains provide a degradation signal for proteasome to eliminate the modified substrates; K63-linked polyubiquitin chains regulate protein trafficking and complex assembly, whereas K11/K48-linked polyubiquitin chains promote the rapid removal of aggregation-prone proteins (10).

Although E3 ligases are the key enzymes that recognize specific substrates for their ubiquitination and several E3 ligases have become important drug targets (11), DUBs also play essential roles in modulating protein ubiquitination and stability, thus maintaining cellular proteostasis. Over 100 DUBs have been discovered in the human genome and are classified into nine subfamilies (12). Among them, the ubiquitin-specific protease (USP) subfamily is the largest group of DUB subfamily, containing more than 50 members. Ubiquitin-specific protease 7 (USP7, also called HAUSP), one of the most extensively studied DUBs, can deubiquitinate MDM2/MDMX and thus enhance their stability to promote p53 ubiquitination and degradation. Therefore, inhibition of USP7 elevates p53, leading to cell cycle arrest and apoptosis (13). USP39 is a USP family DUB which is highly conserved in yeast, zebrafish, mouse, and human (14, 15). Human USP39 contains an arginine-rich (AR) domain at the N terminus, a zinc finger (ZF) domain, and a USP domain at the C terminus. USP39 modulates the deubiquitination of several substrates, including CHK2 (16), cyclin B1 (17), IκBα (18), and STAT1 (19), thereby participating in DNA damage response, cell proliferation, cell apoptosis, antiviral activity, and anti-inflammatory response. Interestingly, USP39 can remove different types of polyubiquitin chains from protein substrates. For example, it deconjugates the K6-linked polyubiquitin chains from STAT1 (19), K29-linked polyubiquitin chains from cyclin B1 (17), and K48-linked polyubiquitin chains from IκBα (18), respectively, and hence enhances their stability. It has also been revealed that USP39 regulates cell proliferation, migration, invasion, and apoptosis of several cancers, including colon cancer (20, 21), glioma (17, 22), and hepatocellular carcinoma (23, 24, 25, 26). Although it has been shown that USP39 has played significant roles in regulating the ubiquitination and stability of multiple proteins, it is unclear whether it impacts RBM39 and regulates its biological functions in gastric cancer cells.

In this work, we first employ affinity purification coupled with label-free quantitative proteomics to identify RBM39-interacting proteins. Then, we screen about 70 DUBs to uncover DUBs that regulate the RBM39 protein level and explore how USP39, an RBM39-interacting DUB, regulates the RBM39 protein level. We further investigate the influence of USP39 on the growth and metastasis of gastric cancer cells and reveal the molecular mechanism by which USP39 executes its biological function and its association with RBM39 expression. This work may provide an alternative strategy for modulating RBM39 for targeted cancer therapy.

## Results

### Affinity purification coupled with quantitative proteomics identifies RBM39-interacting proteins

**Figure 1:**
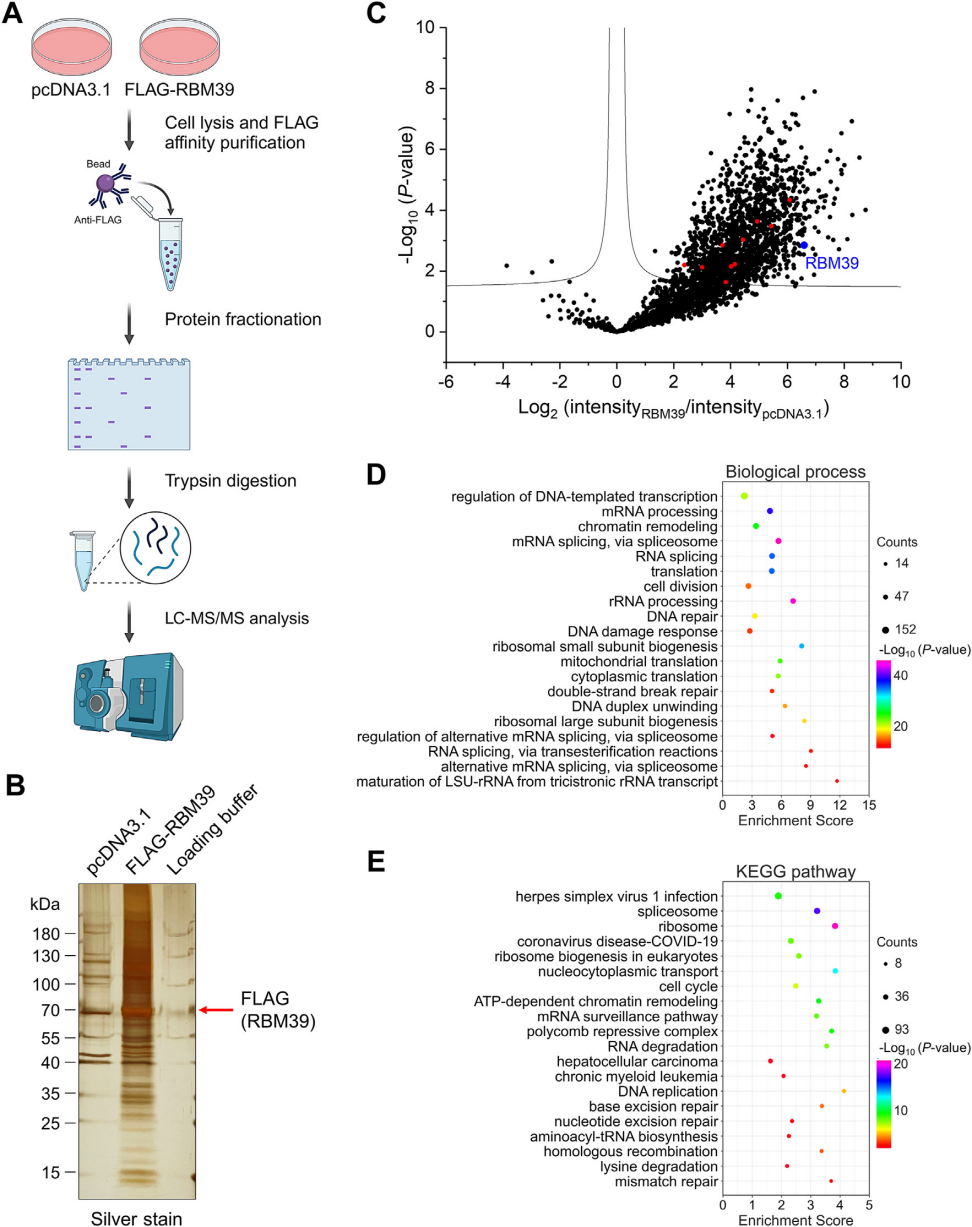
**Proteomic identification of RBM39-interacting proteins.***A*, schematic illustration of the experimental procedure for the identification of RBM39-interacting proteins. HEK293T cells were transfected with pcDNA3.1 or FLAG-RBM39 plasmid for 48 h and lysed in the modified radioimmunoprecipitation assay buffer. FLAG-RBM39 and its interacting proteins were purified by anti-FLAG affinity gel, separated with SDS-PAGE, reduced with dithiothreitol, alkylated with chloroacetic amide, digested with trypsin, desalted with C18 ziptip, and analyzed with mass spectrometry. *B*, silver staining of proteins that were purified by anti-FLAG affinity gel and separated with SDS-PAGE. The *red* arrow indicated FLAG-RBM39. *C*, the volcano plot of RBM39-interacting proteins obtained from three biological replicates of proteomic analysis of anti-FLAG affinity gel purified samples. -Log_10_ (*p* value) and Log_2_ (intensity_RBM39_/intensity_pcDNA3.1_) were obtained from Perseus. The *blue dot* and *red dots* represent RBM39 and deubiquitinating enzymes (DUBs), respectively. *D*–*E*, biological process and Kyoto Encyclopedia of Genes and Genomes pathway analysis of potential RBM39-interacting proteins using DAVID bioinformatics resources (https://david.ncifcrf.gov).

Recently it has been demonstrated that RBM39 is a biomarker for different cancers and regulates alternative splicing in cancer cells (6, 7). It has also been revealed that sulfonamides including indisulam, E7802, tasisulam, and chloroquinoxaline sulfonamide can form tertiary complexes with RBM39 and DCAF15, a substrate receptor of the cullin 4-RING E3 ligase, to promote the ubiquitination and proteasomal degradation of RBM39 (5). However, it is unknown how RBM39 is regulated in gastric cancer cells. To identify the possible upstream regulators for RBM39, we performed an affinity purification and mass spectrometry (MS) analysis. In this experiment, HEK293T cells were transfected with pcDNA3.1 or a plasmid expressing FLAG-RBM39. RBM39 and its interacting proteins were isolated with anti-FLAG affinity gel and digested with trypsin in gel followed by MS analysis (Fig. 1*A*). Silver staining revealed that many protein bands distinct from the mock immunoprecipitate were present in the FLAG-RBM39 immunoprecipitate, with RBM39 as the most intense band (Fig. 1*B*). In total, liquid chromatography tandem-mass spectrometry identified 2603 proteins which have at least two identified tryptic peptides (Table S1). The volcano plot obtained from label-free quantification of MS data revealed that 1641 proteins were significantly enriched in the RBM39 immunoprecipitate and were considered potential RBM39-interacting proteins. Among them, several proteins, such as splicing factor 3B subunit 1 (SF3B1), splicing factor U2AF 65 kDa subunit (U2AF65), E3 ubiquitin/ISG15 ligase TRIM25, DNA damage-binding protein 1 (DDB1), cullin 4A, cullin 4B, and JUN, were previously identified as RBM39-interacting proteins based on the analysis of the curated proteins in the BioGRID database (https://thebiogrid.org) (27), suggesting that this experiment could confidently identify RBM39-interacting proteins. Among them, DDB1 and cullin 4A/B are the components of the cullin 4-RING E3 ligases. In addition, ten identified proteins are DUBs, including BAP1, OTUD4, USP7, USP9X, USP10, USP24, USP36, USP39, USP42, *etc*. (Fig. 1*C*, red circles). Bioinformatic analysis unveiled that the potential RBM39-interacting proteins participated in the regulation of gene transcription, RNA splicing, DNA damage response, and translation (Fig. 1, *D* and *E*). This is in accordance with the previous findings obtained from the high-throughput screening of gene expression and splicing events regulated by RBM39 (6).

### Identification of USP39 as a potential DUB for RBM39

Although the above proteomics analysis unveiled that many RBM39-interacting proteins participate in the ubiquitin-proteasome system (UPS), it cannot determine which proteins indeed regulate the RBM39 protein level. It has been discovered that RBM39 could be degraded through the UPS in the presence of molecular glues such as indisulam. Therefore, we thought to test whether RBM39 could also be regulated by the UPS in the absence of molecular glues. To do so, we treated AGS and MKN45 cells with proteasome inhibitors MG132 and bortezomib, lysosome inhibitors bafilomycin A1 (Baf A1), NH_4_Cl, and chloroquine, and immunoblotted RBM39 in the cell lysates. The result disclosed that MG132 and bortezomib but not Baf A1, NH_4_Cl, and chloroquine significantly increased the RBM39 protein level (Fig. S1), indicating that RBM39 might be degraded through the UPS even in the absence of molecular glues.

**Figure 2:**
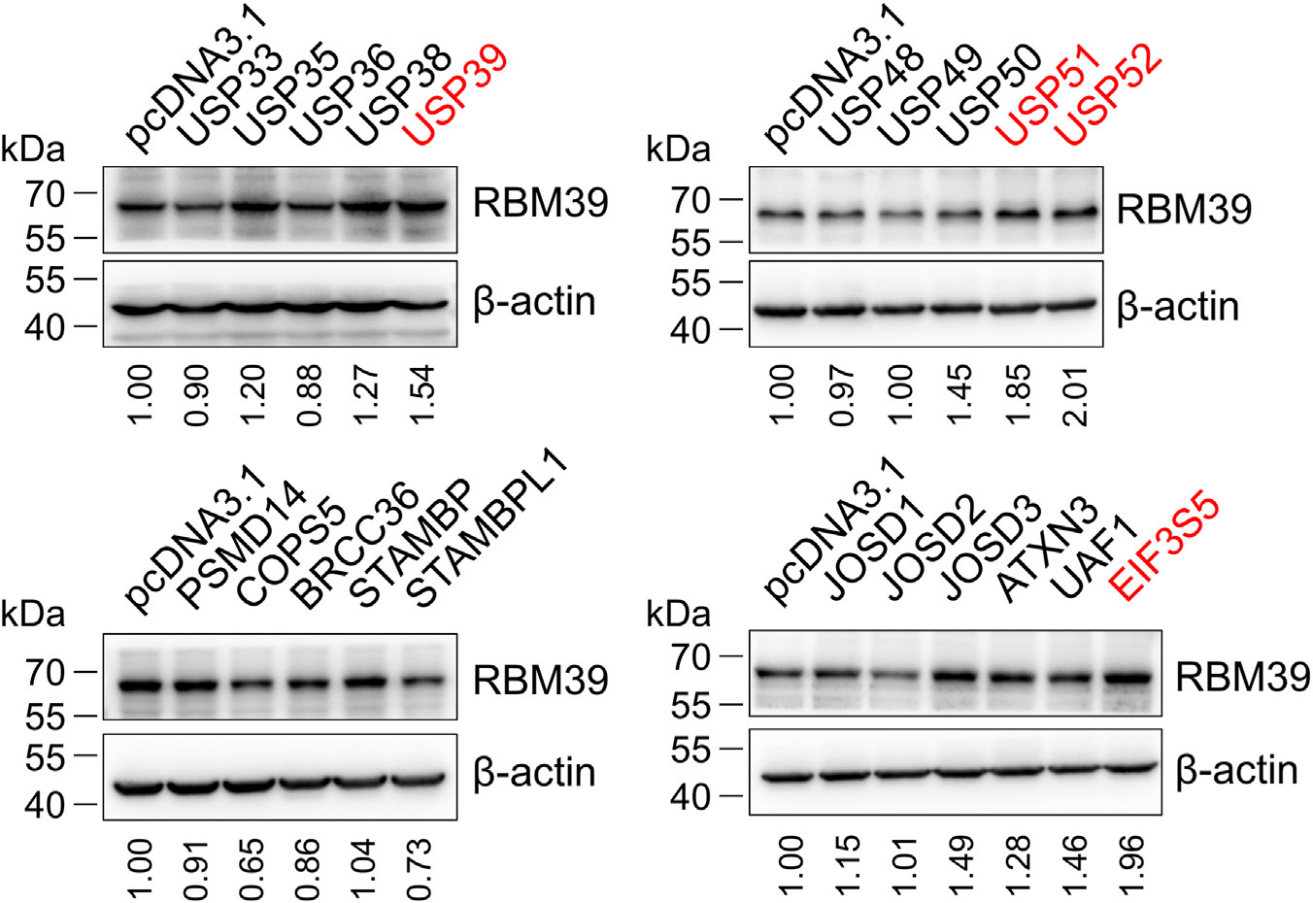
**Biochemical screen of DUBs that regulate RBM39 protein level.** HEK293T cells were transfected with pcDNA3.1 or individual DUB plasmids for 48 h and lysed in the modified radioimmunoprecipitation assay buffer. The resulting cell lysates were immunoblotted for RBM39 and β-actin. The values under images were relative intensities for RBM39 after normalized with β-actin. The DUBs that upregulated RBM39 were indicated by *red text*. Western blotting images for RBM39 after expressing other DUBs in HEK293T cells were provided in Fig. S2. DUB, deubiquitinating enzyme.

Since DUBs could remove the conjugated ubiquitin from protein substrates and hence impair their ubiquitin-mediated degradation, we examined the effect of DUBs on the RBM39 protein level. To identify DUBs that could significantly regulate RBM39, we expressed 69 DUBs and associated proteins available in our laboratory in HEK293T cells and immunoblotted RBM39 in the cell lysates. This midsized screening discovered that several DUBs including USP39, USP51, and USP52 significantly elevated RBM39 protein levels by more than 1.5-folds (Figs. 2 and S2). Among them, USP39 was also identified as an RBM39-interacting protein by quantitative proteomic analysis (Table S1 and Fig. S3). Therefore, we thought that USP39 regulated the RBM39 protein level most probably through directly interacting with RBM39 and modulating its ubiquitination and degradation. In addition, it has also been reported that USP39 regulates alternative splicing (26, 28), which is in concert with the function of RBM39 (7, 29). Hence, in our following experiments, we explored the influence of USP39 on gastric cancer cells and elucidated the underlying molecular mechanism.

### USP39 is elevated in gastric cancer tissues and its elevation correlates with poor patient survival

**Figure 3:**
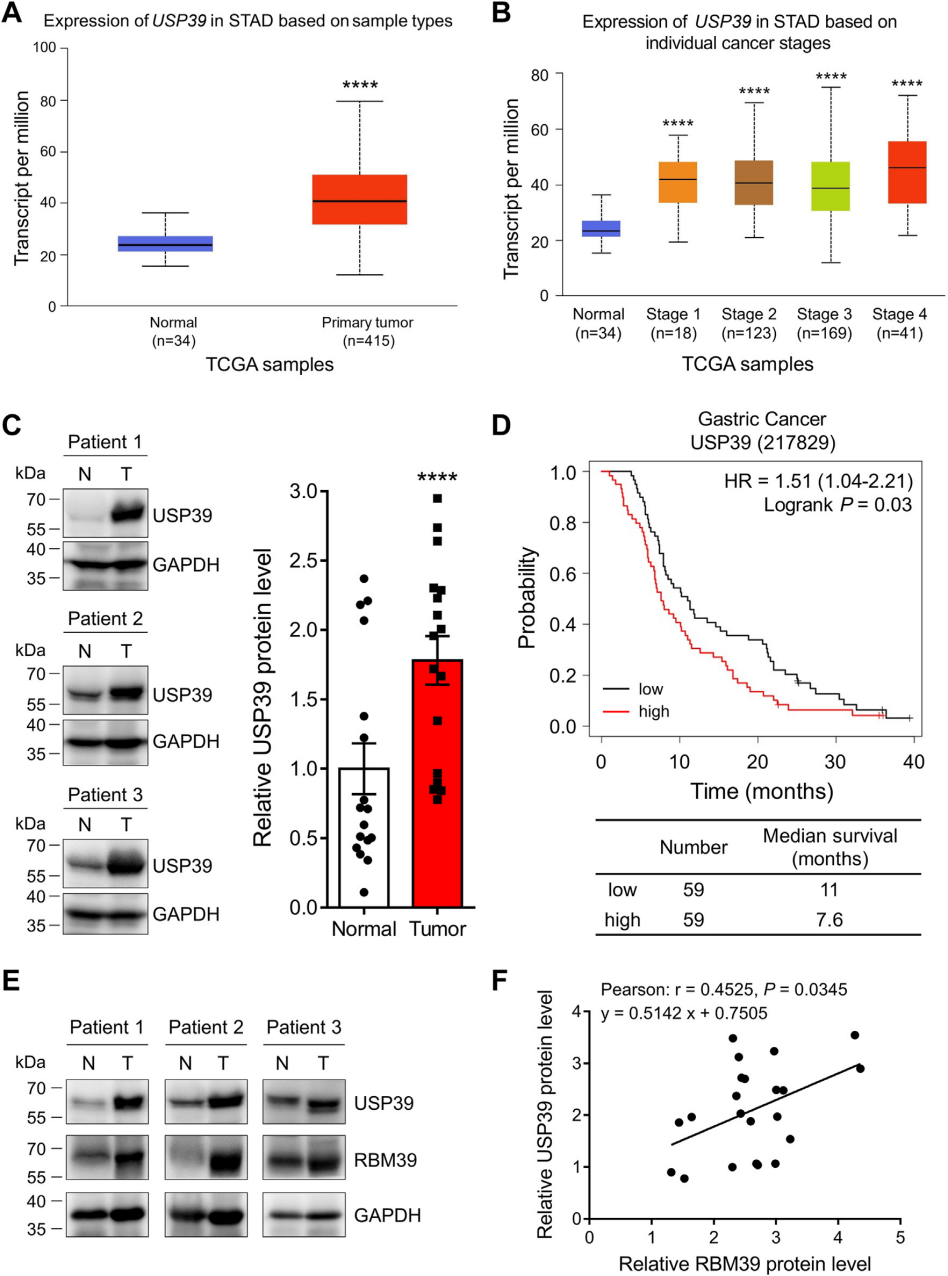
**USP39 is highly expressed in gastric cancer tissues and its high expression is correlated with poor prognosis.***A*–*B*, database analysis reveals that *USP39* mRNA is highly expressed in gastric cancer tissues. *USP39* mRNA level in normal and gastric cancer tissues (*A*) and in gastric cancer tissues at individual cancer stages (*B*) was obtained from the UALCAN database (https://ualcan.path.uab.edu/analysis.html). *C*, USP39 protein is elevated in gastric cancer tissues. Normal tissues adjacent to tumor and tumor tissues obtained from gastric cancer patients were lysed for Western blotting analysis of USP39 (n = 17). *D*, Kaplan–Meier plotter analysis reveals that high *USP39* mRNA expression reduces the survival of gastric cancer patients. Data were obtained from https://kmplot.com/analysis. *E*–*F*, USP39 and RBM39 proteins are positively correlated. *E*, Western blotting analysis of USP39 and RBM39 from tissue lysates of three gastric cancer patients. *F*, correlation between relative RBM39 and USP39 protein levels obtained from Western blotting analysis. Pearson’s correlation coefficient *r* = 0. 4525 and *p* = 0.0345. Mean ± SD, Student’s *t* test with group (*A* and *B*) or paired (*C*) comparisons, ∗∗∗∗: *p* < 0.0001. N, normal; T, tumor.

To explore the function of USP39 on the regulation of gastric cancer cells, we first assessed its mRNA expression in tumor tissues. Analysis of The Cancer Genome Atlas database revealed that *USP39* mRNA was highly expressed in the primary gastric tumor (Fig. 3*A*) and was significantly elevated at the different stages of gastric cancer tissues although the relative level at various stages remained similar (Fig. 3*B*). Second, we validated this result using tissue samples from gastric cancer patients. Immunoblotting of USP39 in the lysates of tumor and paratumor tissues revealed that USP39 protein was indeed highly elevated in gastric tumor tissues (Fig. 3*C*), which is consistent with the *USP39* mRNA expression in tumor tissues obtained from The Cancer Genome Atlas database. Third, Kaplan–Meier survival analysis revealed that high *USP39* mRNA expression results in poor survival of gastric cancer patients (Fig. 3*D*). A similar situation was also discovered for RBM39 expression and patient survival (30). To further confirm the potential correlation between USP39 and RBM39, we immunoblotted these two proteins in different gastric cancer cell lines and in paratumor and tumor tissues from gastric cancer patients. Immunoblotting of lysates from different cell lines resulted in a positive correlation between these two proteins with a high Pearson’s correlation coefficient of 0.83 (Fig. S4). Immunoblotting of tissues clearly indicated the high expression of RBM39 and USP39 in tumor tissues and their protein levels also had a positive correlation with a Pearson’s correlation coefficient of 0.45 (Fig. 3, *E* and *F*). These results suggest that USP39 might regulate RBM39 and its biological function in gastric cancer. It should be noted that other DUBs (such as USP50, USP51, USP52, JOSD3, and UAF1), which were identified to potentially regulate RBM39 in the biochemical screening, neither have high mRNA expression in gastric cancer tissues nor affect the overall patient survival based on the database analysis (Fig. S5). Therefore, we focused on the roles of USP39 in the regulation of RBM39 and its biological functions in this work.

### USP39 interacts and colocalizes with RBM39

**Figure 4:**
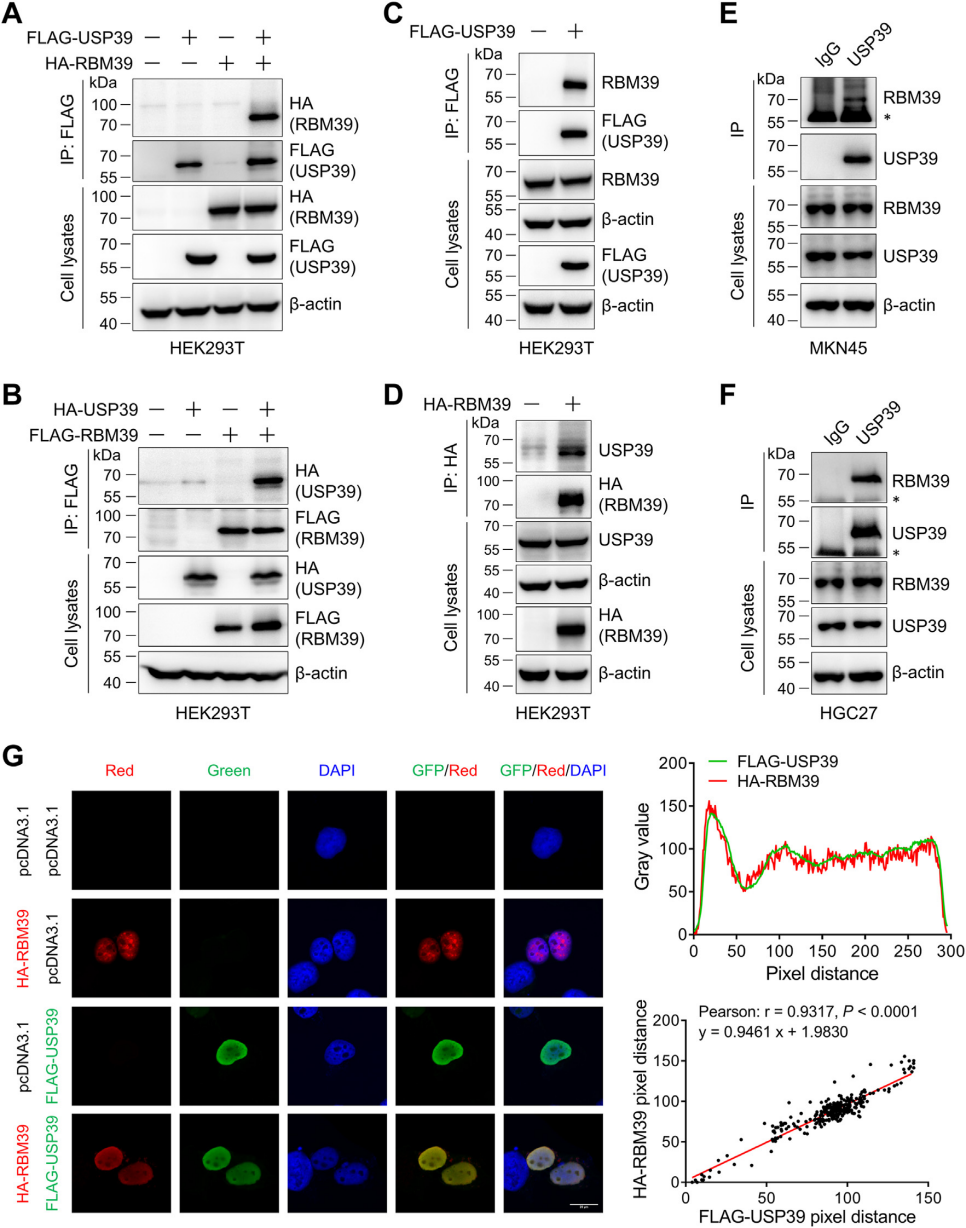
**Biochemical validation of the interaction between USP39 and RBM39.***A*–*B*, exogenous expressed USP39 and RBM39 interact with each other. HEK293T cells were transfected with the indicated plasmids for 48 h and USP39 or RBM39-interacting proteins were immunoprecipitated with anti-FLAG affinity gel. Cell lysates and immunoprecipitates were immunoblotted with the indicated antibodies. *C*–*D*, exogenously expressed USP39 and RBM39 interact with the endogenous RBM39 and USP39. HEK293T cells were transfected with FLAG-USP39 or HA-RBM39 plasmids for 48 h, followed by anti-FLAG or anti-HA immunoprecipitation and Western blotting with the indicated antibodies. *E*–*F*, USP39 interacts with RBM39 endogenously. MKN45 and HGC27 cells were lysed and endogenous USP39 and its interacting proteins were immunoprecipitated with anti-USP39 antibody. Cell lysates and immunoprecipitates were immunoblotted with RBM39 and USP39. IgG was used in the control group. ∗: antibody heavy chain. *G*, RBM39 and USP39 colocalize in the nucleus. HEK293 cells were transfected with HA-RBM39 and/or FLAG-USP39 plasmids for 48 h, washed with ice-cold PBS, fixed with 4% formaldehyde, permeabilized with 0.1% Triton X-100, incubated with FLAG/HA primary antibodies and fluorescent secondary antibodies, stained with DAPI, and photographed under a confocal laser microscope. The scale bar represents 20 μm. Intensity traces from the original images were obtained using the plot profile tool in ImageJ and plotted on the *right side*. Colocalization coefficient was calculated by Pearson’s correlation analysis. *Red*: RBM39; *green*: USP39; *blue*: DAPI. DAPI, 4′,6-diamidino-2-phenylindole; HA, hemagglutinin.

Our proteomics and DUB screening experiments demonstrated that USP39 interacted with RBM39 and upregulated its protein level. Next, we sought to verify their interaction biochemically. First, we performed the coimmunoprecipitation experiments in the USP39 or/and RBM39 overexpressed HEK293T cells. Hemagglutinin (HA) tagged RBM39 (HA-RBM39) was detected in the immunoprecipitate of FLAG-USP39 (Fig. 4*A*) and HA-USP39 was also observed in the reverse immunoprecipitation of FLAG-RBM39 (Fig. 4*B*), indicating their interaction in the overexpressed system. Second, we expressed either FLAG-USP39 or HA-RBM39 in HEK293T cells and immunoprecipitated them with anti-FLAG affinity gel or anti-HA magnetic beads. Immunoblotting of immunoprecipitates specifically detected endogenous RBM39 and USP39, respectively (Fig. 4, *C* and *D*). Third, to test whether this interaction occurs endogenously in gastric cancer cells, we immunoprecipitated endogenous USP39 from MKN45 and HGC27 cell lysates using an anti-USP39 antibody and protein A/G agarose and detected the presence of endogenous RBM39 in the immunoprecipitates (Fig. 4, *E* and *F*). Fourth, we examined whether USP39 and RBM39 colocalize with each other in cells. Immunofluorescence results clearly demonstrated that FLAG-USP39 (green) and HA-RBM39 (red) overexpressed in HEK293 cells were indeed remarkably colocalized in the nucleus with a high Pearson’s correlation coefficient of 0.93 (Fig. 4*G*). Taken together, these results manifested the interaction and colocalization of USP39 and RBM39.

### The N-terminal domain in RBM39 preferentially interacts with the AR and USP domains in USP39

**Figure 5:**
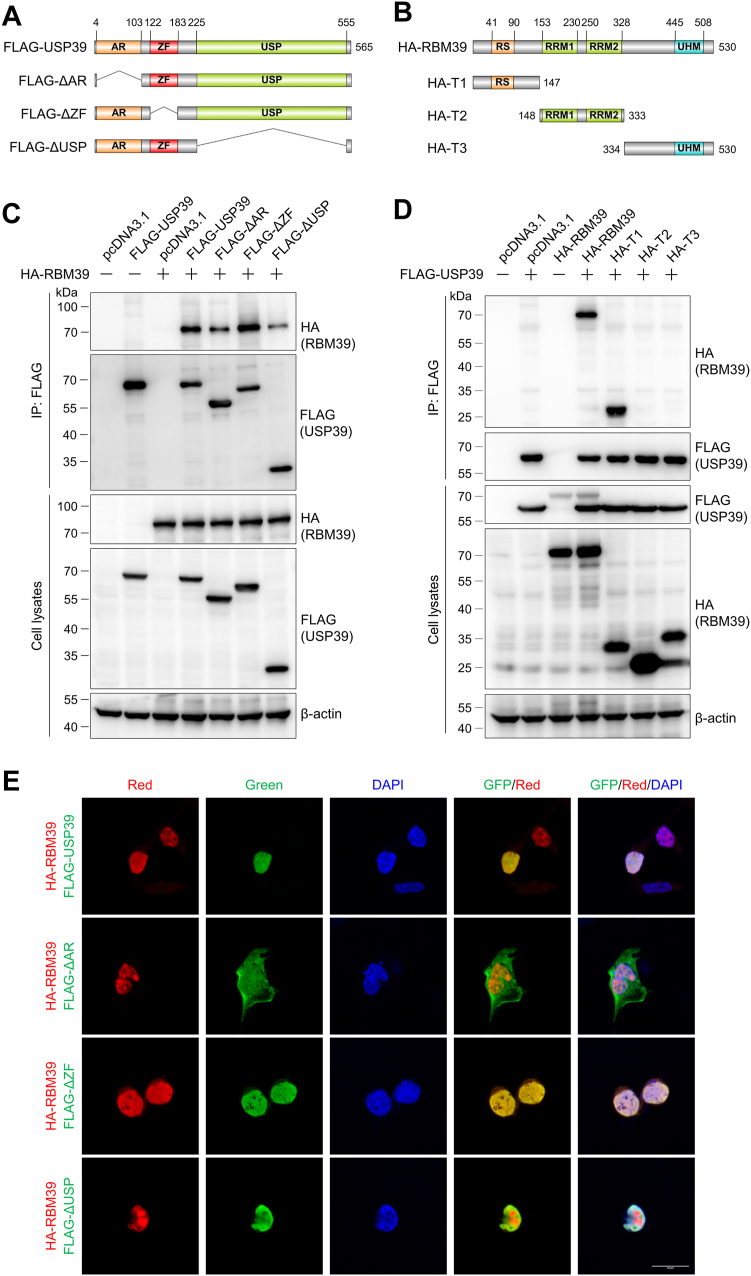
**The N-terminal domain in RBM39 is required for its interaction with USP39.***A*–*B*, illustration of USP39 and its domain deletion mutants (*A*) or RBM39 and its truncation mutants (*B*). FLAG and HA were added to the N terminus of the constructed plasmids. The functional domains were indicated in the diagram. *C*, the AR and USP domains are important for USP39 to interact with RBM39. HEK293T cells were transfected with HA-RBM39 and FLAG-USP39 or its domain deletion mutants for 48 h and lysed in the modified radioimmunoprecipitation assay buffer. Cell lysates were purified with anti-FLAG affinity gel. Cell lysates and the purified samples were analyzed by Western blotting. *D*, the N-terminal domain in RBM39 interacts with USP39. HEK293T cells were transfected with FLAG-USP39 and HA-RBM39 or its truncation mutants for 48 h and treated as described in (*C*). *E*, the interaction between RBM39 and USP39 is important for their localization. HEK293 cells were transfected with HA-RBM39 and FLAG-USP39 or its domain deletion plasmids for 48 h and immunofluorescence experiments were conducted as described in Fig. 4G. The scale bar represents 20 μm. The quantification data for their colocalization were provided in Fig. S6. AR, arginine-rich domain; HA, hemagglutinin; USP, ubiquitin-specific protease domain; ZF, zinc finger domain; RRM, RNA recognition motif; RS, arginine-serine rich domain; UHM, U2AF homology motif.

To determine the interacting domains in RBM39 and USP39, we constructed a series of domain deletion USP39 mutants and truncated RBM39 mutants (Fig. 5, *A* and *B*). We coexpressed the full-length RBM39 with USP39 and its domain deletion mutants in HEK293T cells. Coimmunoprecipitation of RBM39 and its interacting proteins clearly revealed that deletion of the AR domain or the USP domain in USP39 significantly reduced its interaction with RBM39 (Fig. 5*C*), implying that these two domains are critical to maintaining their interplay. In addition, coimmunoprecipitation of USP39 with truncated RBM39 mutants disclosed that USP39 immunoprecipitated the full length and the N-terminal domain (aa 1–147) of RBM39 (Fig. 5*D*). To further explore the effect of their interaction on the colocalization and RBM39 protein level, we expressed RBM39 and USP39 or its domain deletion mutants and conducted the immunofluorescence and immunoblotting experiments. The immunofluorescence images showed that the full-length USP39 and the RBM39-interacting mutant (USP39 ΔZF) were highly colocalized with RBM39, while the USP39 ΔAR and ΔUSP mutants have a much lower degree of colocalization with RBM39 (Figs. 5*E* and S6). In addition, the USP39 ΔAR mutant was mainly localized in the cytoplasm because the nuclear localization sequence in the AR domain was absent in this mutant (Fig. 5*E*). Furthermore, immunoblotting results demonstrated that the full-length USP39 and the USP39 ΔZF mutant could increase the RBM39 protein level, while the other two deletion mutants almost did not affect RBM39 (Fig. S7). These experiments demonstrated that the N-terminal domain in RBM39 and the AR and USP domains in USP39 are important for their interaction and colocalization.

### Upregulation of RBM39 by USP39 requires its enzymatic activity

**Figure 6:**
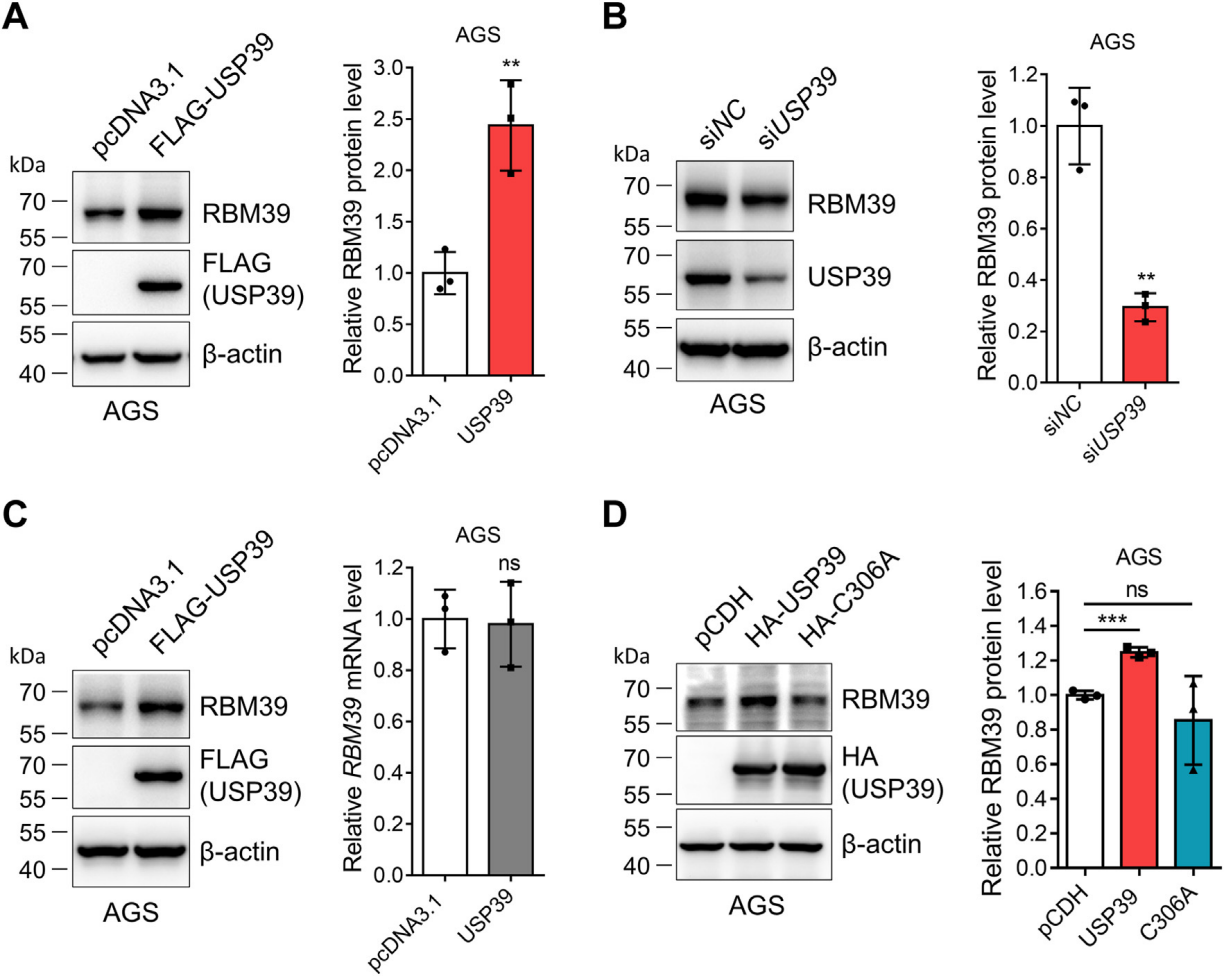
**USP39 upregulates the RBM39 protein level through its deubiquitinating activity.***A*, USP39 expression increased RBM39. AGS cells were transfected with pcDNA3.1 and FLAG-USP39 plasmids, lysed, and immunoblotted with the indicated plasmids. *B*, *USP39* knockdown diminished RBM39. AGS cells transfected with si*NC* or si*USP39* for 48 h were harvested to obtain cell lysates for Western blotting analysis. *C*, USP39 expression did not alter *RBM39* mRNA level. AGS cells were transfected with pcDNA3.1 and FLAG-USP39 plasmids. Proteins and RNAs were extracted with radioimmunoprecipitation assay buffer and TRIzol, respectively. The expression levels of RBM39 protein and mRNA were evaluated by Western blotting and real-time fluorescence quantitative PCR, respectively, after synthesis of the first strand cDNA. *D*, the deubiquitinating activity was required for USP39 to regulate RBM39. AGS cells were transfected with USP39 and its catalytically inactive C306A mutant, lysed, and the cell lysates were immunoblotted. Mean ± SD (n = 3), Student’s *t* test with group comparisons test (*A*–*C*), one-way ANOVA with Dunnett’s multiple comparisons test (*D*), ∗∗: *p* < 0.01, ∗∗∗: *p* < 0.001, and ns, not significant. Note: the difference in the fold change of RBM39 in (*A*) and (*D*) upon USP39 expression may be caused by the different expression levels of USP39 in two experiments.

After we demonstrated the interaction between USP39 and RBM39, we sought to test whether and how USP39 regulates RBM39 in gastric cancer cells. First, we tested the effect of USP39 on RBM39 in AGS gastric cancer cells. Expression of USP39 significantly elevated RBM39 (Fig. 6*A*) while *USP39* knockdown attenuated RBM39 (Fig. 6*B*), which was also observed in HGC27 gastric cancer cells (Fig. S8). However, USP39 expression did not influence the *RBM39* mRNA level (Fig. 6*C*), indicating that the increase of RBM39 by USP39 occurred at the posttranslational level. Second, we asked whether the enzymatic activity of USP39 is required for this upregulation. Expression of the WT USP39 but not the catalytically inactive C306A mutant elevated RBM39 protein level (Fig. 6*D*). Altogether, these results indicated that USP39 upregulated RBM39 through its catalytic function as a DUB.

### USP39 increases the stability of RBM39 by attenuating its ubiquitination and degradation

**Figure 7:**
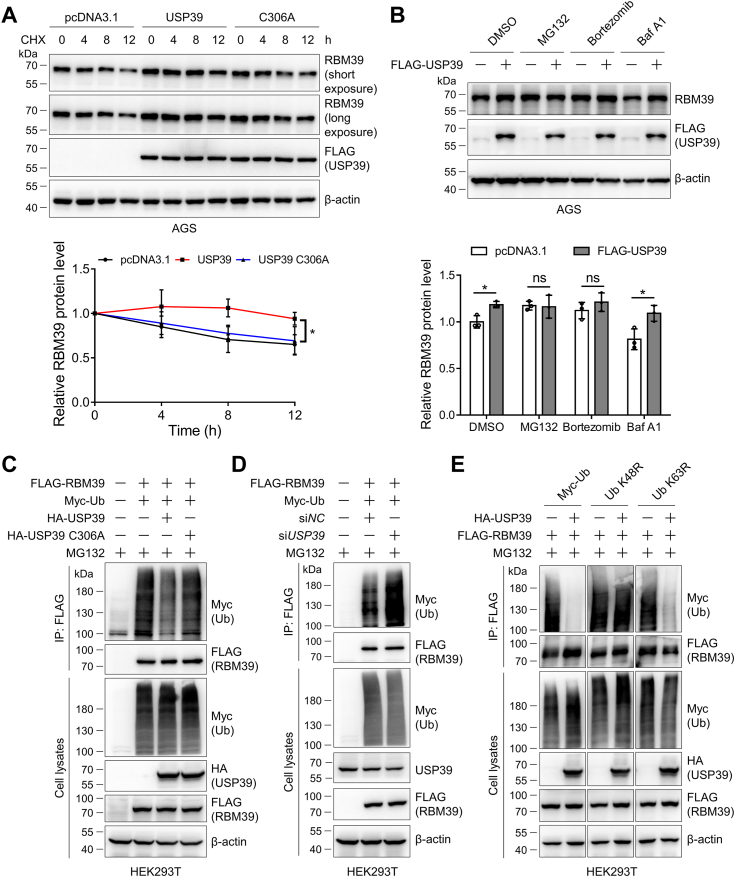
**USP39 stabilizes RBM39 in a proteasome-dependent manner.***A*, USP39 diminishes RBM39 degradation. AGS cells were transfected with pcDNA3.1, FLAG-USP39, or the C306A mutant plasmid for 48 h and split into 6-well plates. Cells were treated with 200 μg/ml cycloheximide (CHX) for different durations, collected, and lysed for Western blotting analysis. The relative RBM39 protein intensity was quantified with ImageJ. *B*, proteasomal inhibition abolishes the effect of USP39 on RBM39. AGS cells were transfected with pcDNA3.1 or FLAG-USP39 plasmid for 48 h and then each was divided into four wells in 6-well plates. Cells were treated with DMSO, 5 μM MG132, 10 μM bortezomib, or 100 nM Baf A1, respectively, for 12 h and lysed. The resulting cell lysates were immunoblotted. *C–E*, USP39 regulates K48-linked polyubiquitination on RBM39. HEK293T cells were transfected with the indicated plasmids or siRNA for 48 h and treated with MG132 (5 μM) for 12 h. RBM39 was immunoprecipitated with anti-FLAG affinity gel for immunoblotting analysis. Mean ± SD (n = 3), Two-way ANOVA with Sidak’s multiple comparisons test (*A*), Student’s *t* test with group comparisons (*B*), ∗: *p* < 0.05, ∗∗: *p* < 0.01, and ns, not significant. Baf A1, bafilomycin A1.

Next, we sought to ask how USP39 elevates the RBM39 protein level in gastric cancer cells through the following experiments. First, we transfected AGS cells with the control, USP39, or the catalytically inactive C306A mutant plasmid, split into 6-well plates, and treated them with cycloheximide (CHX), a protein synthesis inhibitor, to investigate the degradation of RBM39. Immunoblotting of RBM39 from cells treated with CHX for different durations unveiled that USP39 but not the C306A mutant can prolong the half-life of RBM39 (Fig. 7*A*). Second, we treated the above-transfected cells with proteasome inhibitors MG132 and bortezomib and lysosome inhibitor Baf A1 to assess the RBM39 degradation pathway. Immunoblotting of the cell lysates showed that USP39 could increase the RBM39 protein level in the absence of proteasome inhibitors. However, when cells were treated with MG132 or bortezomib, but not Baf A1, RBM39 was significantly increased and USP39 could not elevate RBM39 anymore (Fig. 7*B*). These results demonstrated that USP39 upregulated RBM39 by diminishing its proteasomal degradation. Third, to further confirm whether the enhanced stability of RBM39 is associated with its ubiquitination mediated by USP39, we examined the effect of USP39 on the RBM39 ubiquitination in HEK293T cells after transfecting FLAG-RBM39, HA-USP39 or its C306A mutant, si*USP39*, Myc-Ub or its K48R and K63R mutants. Immunoprecipitation and immunoblotting experiments demonstrated that expression of the WT USP39 but not its C306A mutant could reduce RBM39 ubiquitination (Fig. 7*C*). In contrast, *USP39* knockdown could evidently elevate RBM39 ubiquitination (Fig. 7*D*). In addition, the expression of K48R but not K63R ubiquitin mutant abolished the effect of USP39 on the RBM39 ubiquitination (Fig. 7*E*). These data revealed that USP39 enhanced the stability of RBM39 by eliminating its K48-linked polyubiquitination and attenuating its subsequent proteasomal degradation.

### USP39 promotes the growth and metastasis of gastric cancer cells

**Figure 8:**
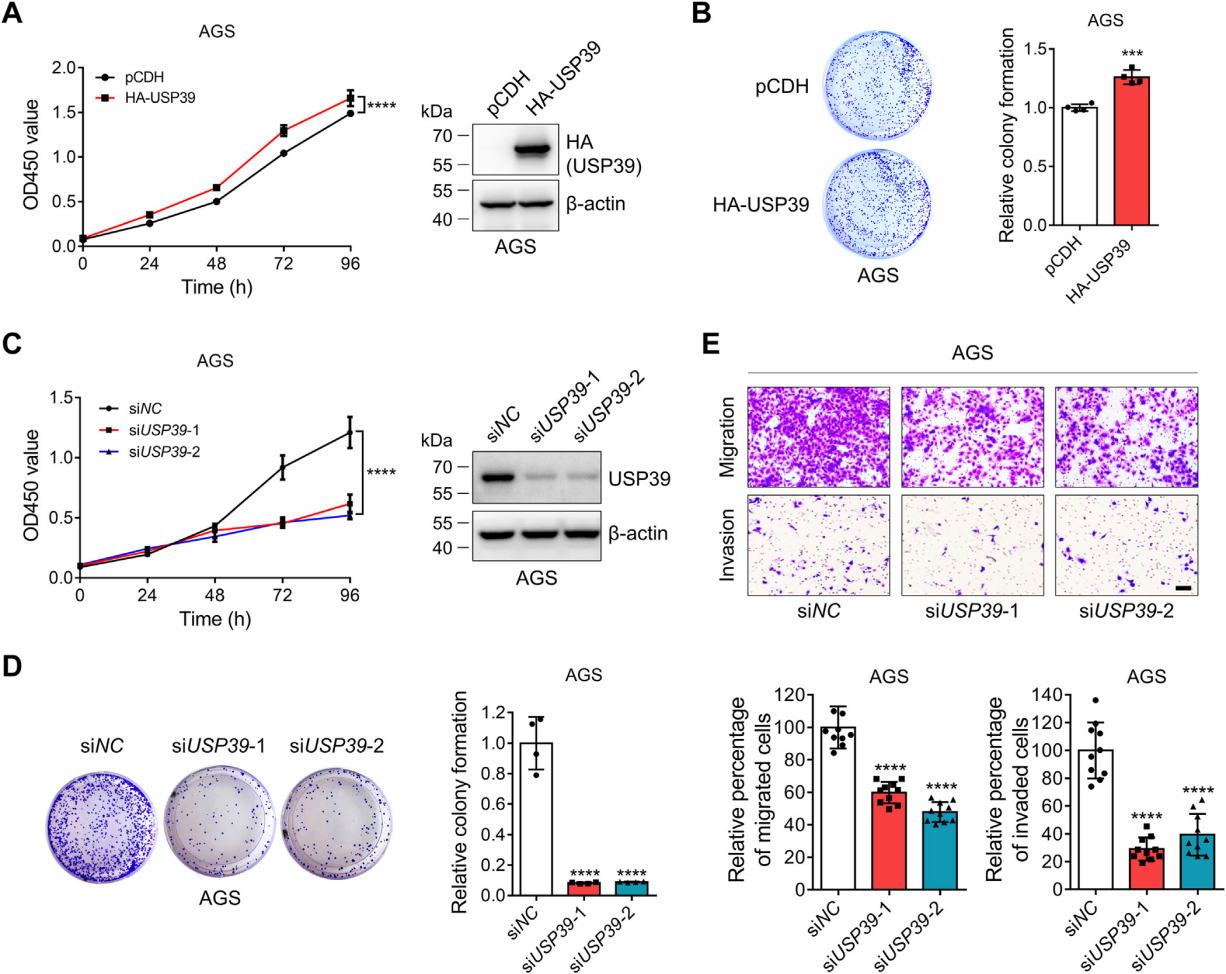
**USP39 facilitates the growth, colony formation, migration, and invasion of gastric cancer cells.***A*-*B*, USP39 expression enhances the growth (*A*) and colony formation (*B*) of gastric cancer cells. AGS cells were transfected with pCDH or pCDH-HA-USP39 plasmid and split into 96-well plates or 35-mm plates for CCK-8 assay and colony formation assay (n = 4). *C-D*, *USP39* knockdown attenuates the growth (*C*) and colony formation (*D*) of gastric cancer cells. AGS cells were transfected with si*NC* or si*USP39* and split into 96-well plates or 35-mm plates for CCK-8 assay and colony formation assay (n = 4). (E) *USP39* knockdown reduces the migration and invasion of gastric cancer cells. AGS cells were transfected with si*NC* or si*USP39* for 24 h and 6 × 10^4^ AGS cells were inoculated into a Transwell chamber or matrigel-coated chamber for migration and invasion assays (n = 10). The scale bar represents 100 μm. Two-way ANOVA with Sidak’s (*A*) or Dunnett’s (*C*) multiple comparisons test, Student’s *t* test with group comparisons (*B*), one-way ANOVA with Dunnett’s multiple comparisons test (*D*–*E*), ∗∗∗: *p* < 0.001 and ∗∗∗∗: *p* < 0.0001. CCK-8, cell counting kit-8.

Our previous work discovered that RBM39 promoted the growth of gastric cancer cells (30) and this work revealed that USP39 upregulated RBM39. Therefore, we would like to test whether USP39 also impacts the growth of gastric cancer cells. Cell counting kit-8 (CCK-8) and colony formation experiments revealed that USP39 overexpression increased (Fig. 8, *A* and *B*), while *USP39* knockdown attenuated (Fig. 8, *C* and *D*) the viability and colony formation of AGS cells. A similar phenomenon was also discovered in the USP39-depleted MKN45 cells (Fig. S9). In addition, *USP39* knockdown significantly reduced the migration and invasion of AGS cells (Fig. 8*E*). These results are consistent with the effect of RBM39 elevation in regulating the growth and colony formation of gastric cancer cells, suggesting that USP39 might execute its biological function by regulating RBM39.

### USP39 executes its biological function partially through RBM39

**Figure 9:**
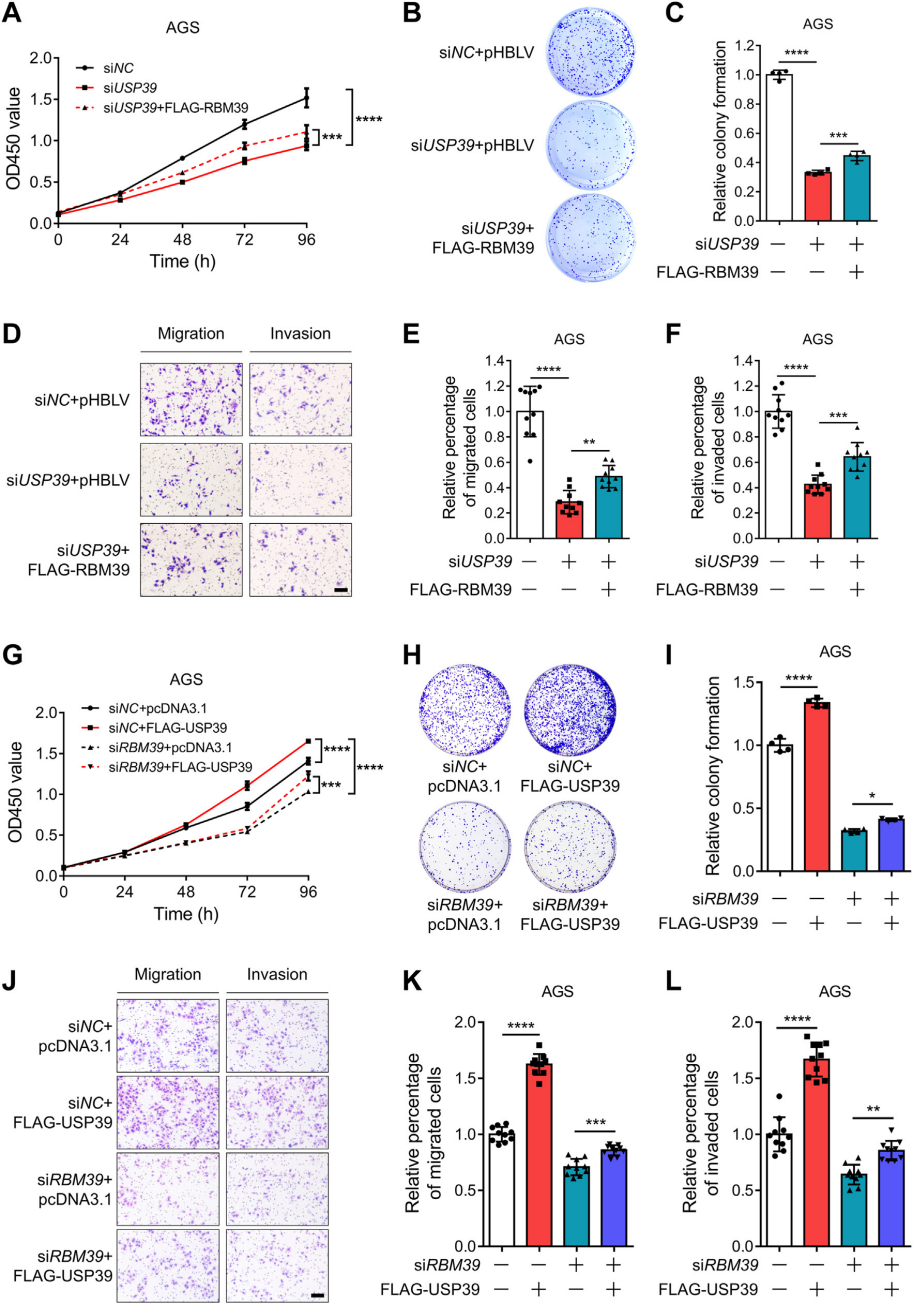
**USP39 promotes the growth, colony formation, migration, and invasion of gastric cancer cells partly through RBM39.***A*–*C*, RBM39 expression partially rescues the effect of *USP39* knockdown on the growth (*A*) and colony formation (*B*–*C*) of gastric cancer cells. AGS cells were transfected with the indicated siRNA and RBM39 plasmid and split into 96-well plates or 35-mm plates for CCK-8 or colony formation assay (n = 4). The transfected cells were lysed and immunoblotted for the indicated antibodies (Fig. S10*A*). *D*–*F*, RBM39 expression partially rescues the effect of *USP39* knockdown on the migration and invasion of gastric cancer cells. AGS cells were treated as described in (*A*) for Transwell migration and invasion assay (n = 10). The scale bar represents 100 μm. *G*-*I*, *RBM39* knockdown partially eliminates the effect of USP39 expression on the growth (*G*) and colony formation (*H*–*I*) of gastric cancer cells. AGS cells were transfected with the indicated siRNA and plasmids and split into 96-well plates or 6-well plates for CCK-8 and colony formation assay (n = 4). The transfected cells were lysed and immunoblotted for the indicated antibodies (Fig. S10*B*). *J*–*L*, *RBM39* knockdown partially abolishes the effect of USP39 expression on the migration and invasion of gastric cancer cells. AGS cells were treated as described in (*G*) for Transwell migration and invasion assays (n = 10). The scale bar represents 100 μm. Two-way ANOVA (*A* and *G*), one-way ANOVA with Tukey’s multiple comparisons test (*C*, *E*, *F*, *I*, *K*, and *L*), ∗: *p* < 0.5, ∗∗: *p* < 0.01, ∗∗∗: *p* < 0.001, and ∗∗∗∗: *p* < 0.0001. CCK-8, cell counting kit-8.

The above work and previous publications (30) demonstrated that both USP39 and RBM39 enhance the growth, colony formation, migration, and invasion of gastric cancer cells. In addition, USP39 elevates RBM39 through its deubiquitinating activity. Therefore, we asked whether USP39 modulates these functions in gastric cancer cells through RBM39. To test this conjecture, we conducted the following experiments. We knocked down *USP39* and transfected RBM39 to restore its expression in AGS cells. CCK-8 assays uncovered that *USP39* knockdown significantly reduced cell viability and further expressing RBM39 partially restored the viability of AGS cells (Figs. 9*A* and S10*A*). Similarly, *USP39* knockdown reduced the colony formation, migration, and invasion of AGS cells, and restoration of RBM39 expression partially rescued the inhibitory effect by *USP39* knockdown (Fig. 9, *B*–*F*). To further validate the effect of USP39 on the function of RBM39, we performed similar experiments in AGS cells with *RBM39* knockdown and USP39 expression (Figs. 9, *G*–*L* and S10*B*). Cell viability, colony formation, migration, and invasion of AGS cells were significantly increased when USP39 was expression but reduced when *RBM39* was knocked down. However, *RBM39* knockdown significantly abolished the effect of USP39 expression on the viability, colony formation, migration, and invasion of AGS cells. Collectively, these results demonstrate that USP39 exhibits its biological functions, at least partially, through downregulating RBM39.

## Discussion

**Figure 10:**
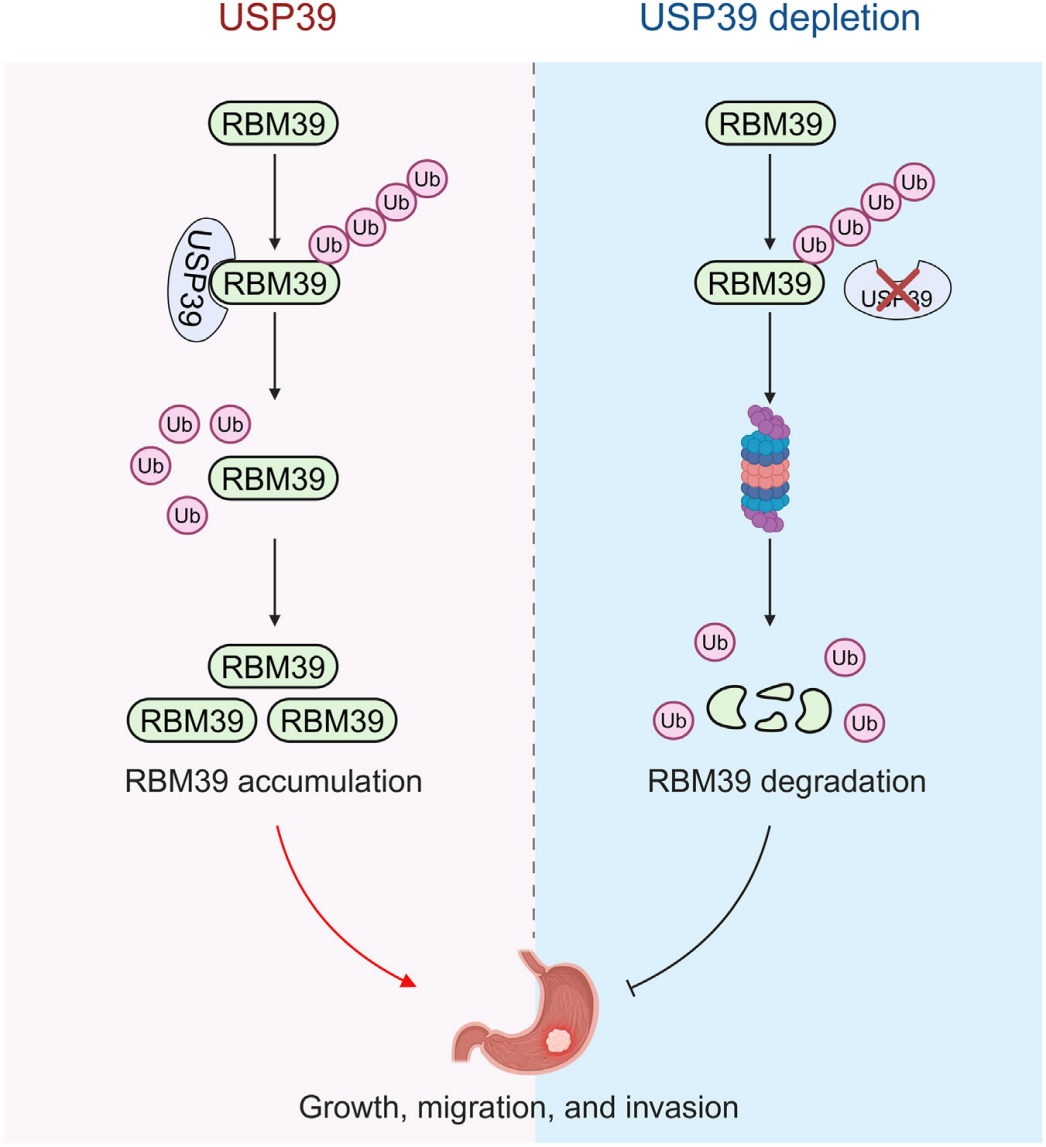
**Proposed model for the influence of USP39 on the growth, migration, and invasion of gastric cancer cells by partially enhancing RBM39 stability.** In the presence of USP39, the conjugated polyubiquitin chains on RBM39 are removed by USP39 and thus RBM39 is accumulated, leading to the growth, migration, and invasion of gastric cancer cells. Upon USP39 depletion, the ubiquitination of RBM39 is enhanced and RBM39 is degraded by the proteasome, resulting in the inhibition of the growth, migration, and invasion of the gastric cancer cells.

The expression of RNA-binding proteins (RBPs) could be regulated at both the genetic and protein levels. Approaches regulating RBPs and their functions include genetic depletion with siRNAs and shRNAs, pharmacological inhibition by small molecules, and protein knockdown using molecular glues, proteolysis-targeting chimeras (PROTACs), or RNA-PROTACs (31). It has been shown that several types of cancer tissues express high *RBM39* mRNA, which is associated with poor patient survival (4). It has also been discovered that RBM39 could be degraded by sulfonamides such as indisulam, E7820, and tasisulam through recruiting DCAF15 to promote its ubiquitination and proteasomal degradation. However, it is unknown how RBM39 is regulated under physiological conditions or in the diseased states. In this work, we revealed that proteasome inhibitors but not lysosome inhibitors could accumulate RBM39 protein and therefore, we hypothesized that the UPS may modulate RBM39 protein levels. Two screening approaches at the protein level, high-throughput quantitative proteomics, and midsized biochemical screening were used to identify the DUBs which could interact with and upregulate RBM39. Affinity purification and quantitative proteomics identified the RBM39-interacting proteins including DUBs, while biochemical approaches further evaluated the effect of USP39 on the RBM39 protein level. Ubiquitination assay also unveiled that USP39 could remove the K48-linked polyubiquitin chains from RBM39, thus enhancing its stability by diminishing its ubiquitination and proteasome degradation. Taken together, this work and previous data (30) revealed that both USP39 and RBM39 promoted the growth of gastric cancer cells and their high expression was associated with poor patient survival. Our work also demonstrated that USP39 accelerated the growth and metastasis of gastric cancer cells partially through RBM39 (Fig. 10). Therefore, inhibition of USP39 might fine-tune the RBM39 protein level and may be an alternative approach for targeted therapeutics. Indeed, it has been revealed that the genetic depletion of USP39 suppressed the proliferation and growth of gastric cancer cells (32, 33).

The UPS is one of the most important pathways for protein degradation and its components were targeted for drug discovery for various diseases, including cancer and neurodegenerative diseases (12). Previous work has revealed that targeting DUBs is a promising approach for cancer therapy and many inhibitors for DUBs have been developed (34). Besides USP39, our proteomic analysis also identified ten other DUBs, including BAP1, OTUD4, USP7, USP9X, USP10, USP24, USP36, USP39, USP42, *etc*., which potentially interact with RBM39. Although our work confirmed that USP39 is a DUB for RBM39, we did not evaluate the effect of the rest of DUBs on the regulation of biological functions of RBM39 since our biochemical screening did not detect a significant reduction in RBM39 protein after expressing other RBM39-interacting DUBs. In addition, our proteomics analysis and biochemical screenings were carried out in HEK293T cells. Hence, we could not completely exclude the possibility that other DUBs may regulate RBM39 in different cell types if their interaction and regulation depend on the presence of other proteins or specific posttranslational modifications. Therefore, our work may not identify all the DUBs that modulate RBM39 stability under different circumstances.

Although our biochemical experiments focused on the DUBs in the RBM39-interacting proteins, our proteomic analysis also detected many E3 ubiquitin ligases such as HUWE1, MID1, TRIM71, UHRF1, WWP1, ZNF92, and ZFP62, as well as multiple subunits of E3 ligase complexes including CUL4A, CUL4B, DDB1, FBXW4, and KEAP1. E3 ligases promote protein ubiquitination and may regulate their stability or other molecular functions such as subcellular localization and protein interaction. For example, KEAP1, a substrate receptor for the cullin 3–RING E3 ligase complex promotes the ubiquitination and degradation of several proteins such as Nrf2 (35) and PD-L1 (36), thus regulating cancer cell growth. Therefore, elevation or activation of E3 ligases may promote the ubiquitination and subsequent degradation of the substrates and thus regulate their biological functions. Indeed, molecular glues and PROTACs have been developed for targeted protein degradation (37, 38, 39). For example, indisulam and other sulfonamides were discovered to target RBM39 and RBM23 for ubiquitination and degradation (5, 40). Besides the proteins in the UPS, we also detected many enzymes, such as AURKB, CDK12, MARK2, TBK1, EZH2, KMT2A, METTL25, SETD2, BRD7, and SENP1, among the RBM39-interacting proteins. These proteins may also alter the stability and biological functions of RBM39 by modulating posttranslational modifications such as phosphorylation, methylation, acetylation, and SUMOylation. Recently, it has been discovered that SENP1 can deSUMOylate the nuclear RBP TDP-43, alter its nucleocytoplasmic distribution, and may modulate its splicing function (41).

This work used both proteomic and biochemical approaches to identify DUBs that regulate the RBM39 protein level. Therefore, the combination of these two approaches identified proteins that most probably directly regulate RBM39. However, this strategy also has its limitations. First, although we identified the key interacting domains in USP39 and RBM39, this work did not identify the key amino acids responsible for their interaction. Second, due to the autoregulation of RBM39 on its mRNA splicing, we are unable to validate the molecular mechanism in animal models using stable *RBM39* knocking down or expressing cell lines. Third, this strategy cannot identify DUBs that modulate other biological functions (such as subcellular localization and protein interaction) of RBM39 but not its protein level. Fourth, we could not explore whether or how other RBM39-interacting proteins regulate its protein level although it has been reported that some of these proteins regulate the subcellular localization and biological functions of RBPs. Nevertheless, we have provided a list of RBM39-interacting proteins that may regulate its biological functions, which could be a useful resource for the RBM39 research community.

In summary, this work utilized quantitative proteomics and biochemical approaches to identify and validate USP39 as a DUB for RBM39, thereby upregulating its protein level by attenuating its ubiquitination and degradation. We also elucidated the molecular mechanism by which USP39 promotes the growth and metastasis of gastric cancer cells partially by regulating RBM39. This work may provide an alternative strategy to targeting RBM39 for proteasomal degradation by inhibiting its DUBs.

## Experimental procedures

### Collection of clinical samples

Gastric cancer tissues were collected with the permission of the Ethics Committee of the First Affiliated Hospital of Soochow University. Cancer tissues and paratumor tissues were excised and homogenized in the modified radioimmunoprecipitation assay buffer to obtain whole tissue lysates for immunoblotting analysis.

### Reagents and antibodies

Reagents and antibodies utilized in this work were provided in Table S2.

### Cell culture and drug treatment

HEK293, HEK293T, and gastric cancer cell lines (AGS, HGC27, and MKN45) were ordered from ATCC and Beijing Beina Chuanglian Biotechnology Research Institute, respectively. Gastric cancer cell lines were confirmed by short tandem repeat profiling. Cells were cultured in high-glucose Dulbecco's modified Eagle's medium (HEK293, HEK293T, and AGS) or RPMI 1640 (HGC27 and MKN45) containing 10% or 20% fetal bovine serum (FBS) and streptomycin/penicillin under a fully humidified incubator with 5% CO_2_ at 37 °C.

For the CHX-chase experiments, cells were seeded in 6-well plates after transfection, treated with CHX (200 μg/ml) for different durations, lysed, and subjected to immunoblotting. For the protein degradation experiments, cells were treated with MG132 (5 μM), bortezomib (10 μM), or Baf A1 (100 nM) for 12 h and the resulting cell lysates were immunoblotted.

### Plasmid construction and transfection

Human *USP39* and *RBM39* complementary DNAs were cloned to pCDH with 3×HA tag at the N terminus, pcDNA3.1 with FLAG tag at the N terminus, or pHBLV with 3×FLAG tag at the C terminus according to a previously described method (42). The complementary DNAs were subcloned to obtain plasmids expressing the truncated USP39 or RBM39. Plasmids were transfected into cells with PEI (408727, Sigma-Aldrich) or lipofectamine 3000 (L3000015, Thermo Fisher Scientific) as described previously (30).

siRNAs-targeting human *USP39* and *RBM39* (Table S3) were synthesized by RiboBio Co and transfected with riboFECT CP transfection reagent (C10511, RiboBio Co) based on a previously published method (43).

### Cell viability and colony formation assays

For cell viability experiments, gastric cancer cells were transfected with plasmids or si*USP39*, split into 96-well plates, and cultured for different durations. CCK-8 was employed to measure cell viability as described previously (44).

For colony formation assay, gastric cancer cells were transfected with plasmids or siRNAs and seeded into 35-mm plates (2000 cells/well). Cells were grown for 10 to 14 days, fixed with 4% paraformaldehyde (in PBS), and stained with 0.1% crystal violet (in PBS). Colonies were photographed and quantified using ImageJ (http://imagej.net).

### Migration and invasion assays

Cell migration and invasion were carried out in Transwell by following a previous method (43). The gastric cancer cells transfected with the indicated plasmids or siRNAs were plated into the upper chambers without (for migration) or with (for invasion) Matrigel matrix (354248, Corning) using an FBS-free culture medium. The lower chambers were filled with 600 μl medium containing 10% FBS. After 36 h of incubation, cells were fixed and stained with 0.1% crystal violet for visualization under a microscope. The migrated or invaded cells were counted with ImageJ for statistical analysis.

### Immunoprecipitation

Immunoprecipitation was performed according to previous procedures (45). Plasmids expressing FLAG-tagged/HA-tagged proteins or/and domain deletion USP39/truncated RBM39 were transfected into HEK293T cells for 48 h. After cells were lysed in the modified radioimmunoprecipitation assay buffer, immunoprecipitation was carried out using anti-FLAG M2 affinity gel (20584ES08, Yeasen) or anti-HA magnetic beads (B26201, Selleck) following the manufacturer’s instructions. Proteins were eluted with buffer containing FLAG peptide (200 μg/ml, DYKDDDDK, 20571ES11, Yeasen) or 2×sample loading buffer. Endogenous USP39 and its interacting proteins from HGC27 and MKN45 cell lysates were immunoprecipitated with anti-USP39 antibody and protein A/G agarose (36403ES08, Yeasen) and eluted by heating in the 2×sample loading buffer.

### Western blotting and silver staining

Western blotting analysis was performed according to a previous method (45). Lysates from cells or tissues and immunoprecipitates were mixed with an appropriate amount of 5×sample loading buffer, heated, centrifuged, and separated by SDS-PAGE. Sequentially, proteins were transferred to PVDF membranes, which were blocked with 5% milk and incubated with the corresponding primary and secondary antibodies. Protein bands were visualized with a Super ECL chemiluminescent substrate under a Tanon 5200 imaging system.

For silver staining, immunoprecipitated samples were subjected to SDS-PAGE, fixed, sensitized, stained, developed, and imaged using the ChemiDoc XRS system (Bio-Rad) according to a previously described method (46).

### Immunofluorescence

Immunofluorescence was performed based on previously described protocols (47, 48). HEK293 cells were transfected with plasmids expressing FLAG-USP39, its domain deletion mutants, or/and HA-RBM39 for 48 h, fixed, permeabilized, blocked, and incubated with the anti-FLAG or anti-HA antibodies, respectively. Cells were further stained with Alexa Flour 594 mouse anti-rabbit IgG and 488 donkey anti-rabbit IgG as well as 4′,6-diamidino-2-phenylindole (DAPI). Images were captured under the A1R HD25 microscope (Nikon). ImageJ was used to analyze the colocalization for RBM39 and USP39 or its domain deletion mutants.

### Quantitative real-time PCR

AGS cells were transfected with pcDNA3.1 or FLAG-USP39 for 48 h. TRIzol reagent was used to extract total RNA. The cDNA library was constructed using 5×All-In-One RT MasterMix (G490, ABM). Real-time fluorescence quantitative PCR was performed using the specific primers (Table S4) and ChamQ Universal SYBR real-time fluorescence quantitative PCR Master Mix (Q711-02, Vazyme) in a CFX Duet real-time PCR system (Bio-Rad).

### Sample preparation and MS analysis

Proteins purified with anti-FLAG affinity gel were prepared and analyzed in an MS instrument as previously described (49, 50). The control and experimental samples from three biological replicates were separated by SDS-PAGE. Gel slices were excised and proteins were reduced, alkylated, and digested with trypsin in polyacrylamide gel. The tryptic peptides were extracted, desalted, and analyzed in an Orbitrap Fusion Tribrid mass spectrometer and data were processed with a method described previously (51). Proteins with at least two identified tryptic peptides were used for quantification. Label-free quantification was performed to acquire the relative abundance of immunoprecipitated proteins. *p* values were calculated using the two-tailed Student’s *t* test. Log_2_ (intensity_RBM39_/intensity_pcDNA3.1_) and -Log_10_ (*p* value) were used to obtain the volcano plot.

### Statistical analysis

Statistical analyses were carried out with GraphPad Prism (http://www.graphpad.com). Statistics for data from two groups were analyzed with Student’s *t* test. One-way ANOVA with Dunnett’s or Tukey’s post hoc test was employed for data from three or more groups. Two-way ANOVA with Tukey’s, Dunnett’s, or Sidak’s post hoc test was utilized for data from CCK-8 assays. Data were presented as mean ± SD. ∗: *p* < 0.05, ∗∗: *p* < 0.01, ∗∗∗: *p* < 0.001, ∗∗∗∗: *p* < 0.0001, and ns, not significant.

## Data availability

The MS proteomics data have been deposited to the ProteomeXchange Consortium (https://proteomecentral.proteomexchange.org) *via* the iProX partner repository (52, 53) with the dataset identifier PXD053963. Other data were incorporated in the main text or supporting materials.

## Supporting information

This article contains supporting information.

## Ethics

All experiments using human specimens in this work were approved by the Ethics Committee of the First Affiliated Hospital of Soochow University.

## Conflict of interest

The authors declare that they have no conflicts of interest with the contents of this article.
