# Supplementary material for: Deubiquitinating enzyme USP39 promotes the growth and metastasis of gastric cancer cells by modulating the degradation of RNA-binding protein RBM39
Source: J Biol Chem. 2024 Sep 10;300(10):107751. doi: 10.1016/j.jbc.2024.107751 (PMC11490714; doi:10.1016/j.jbc.2024.107751)
Supplement: Supporting Information [file mmc1.pdf]

Supporting Figures

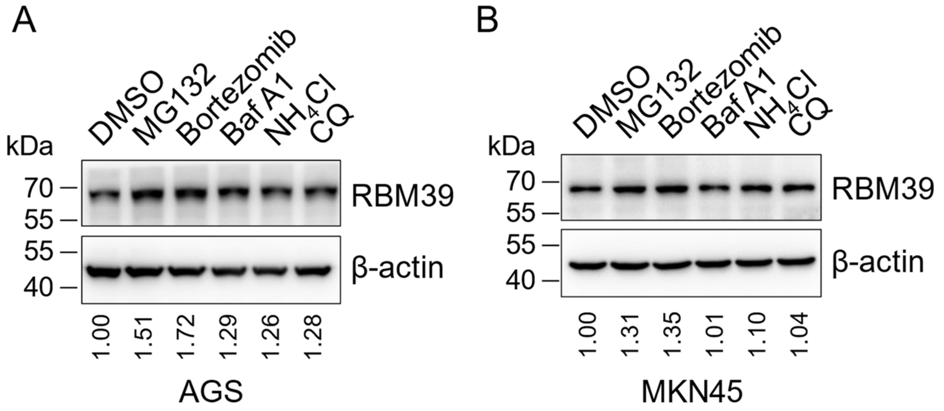

Figure S1. RBM39 is degraded through the proteasomal pathway.

(A) AGS cells were treated with DMSO, 5 µM MG132, 10 µM bortezomib, 100 nM bafilomycin A1 (Baf A1), 10 mM NH_4Cl, or 50 µM chloroquine (CQ) for 10 h. Cells were lysed for Western blotting analysis. The relative intensity of RBM39 was indicated below the images. (B) MKN45 cells were treated with DMSO, 1 µM MG132, 1 µM bortezomib, 100 nM Baf A1, 10 mM NH_4Cl, or 50 µM CQ for 12 h. The cell lysates were immunoblotted with the indicated antibodies. The relative intensity of RBM39 was indicated below the images.

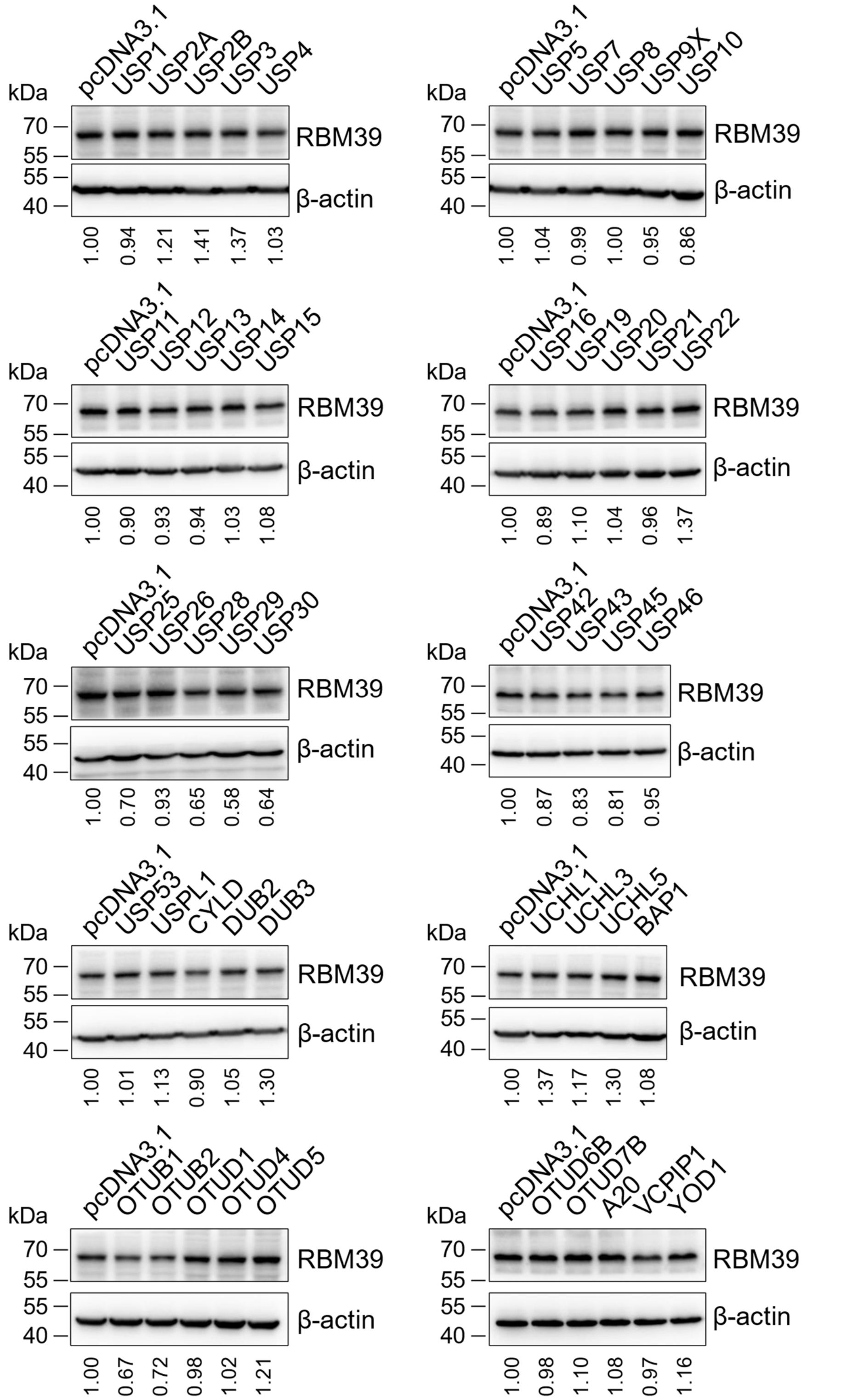

Figure S2. Screening the deubiquitinating enzymes (DUBs) for RBM39 by expressing DUBs in HEK293T cells and immunoblotting the cell lysates.

HEK293T cells were transfected with DUB plasmids individually for 48 h and lysed. The cell lysates were immunoblotted for RBM39 and β-actin. The relative intensity of RBM39 was indicated below the images.

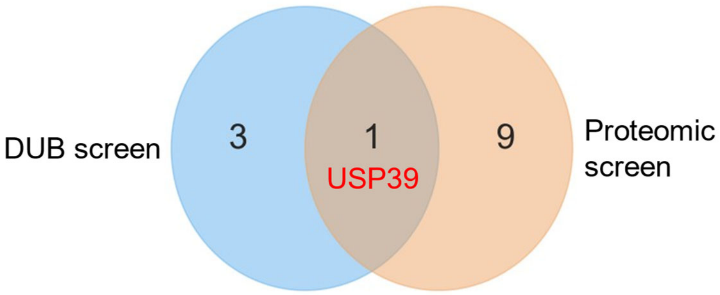

Figure S3. Venn diagram analysis of DUBs obtained from the DUB screening and proteomic analysis of RBM39-interacting proteins.

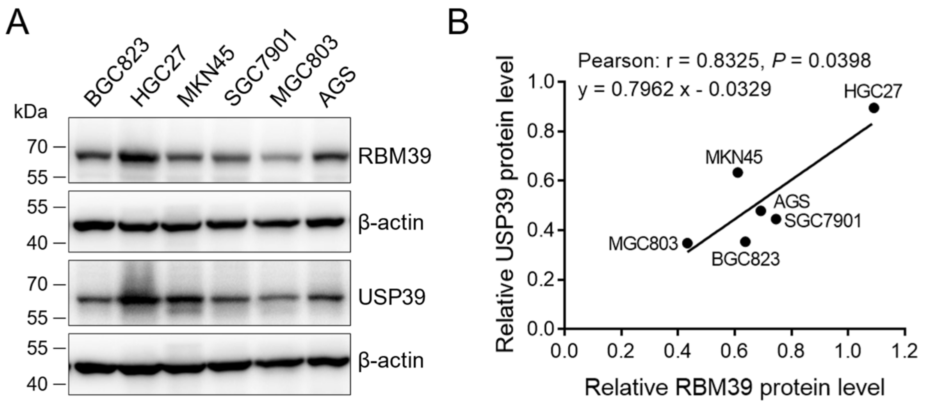

Figure S4. Expression of USP39 and RBM39 protein in gastric cancer cell lines.

(A) Immunoblotting of USP39 and RBM39 proteins in cell lysates obtained from various gastric cancer cell lines. (B) Correlation analysis of USP39 and RBM39 protein levels in gastric cancer cell lines.

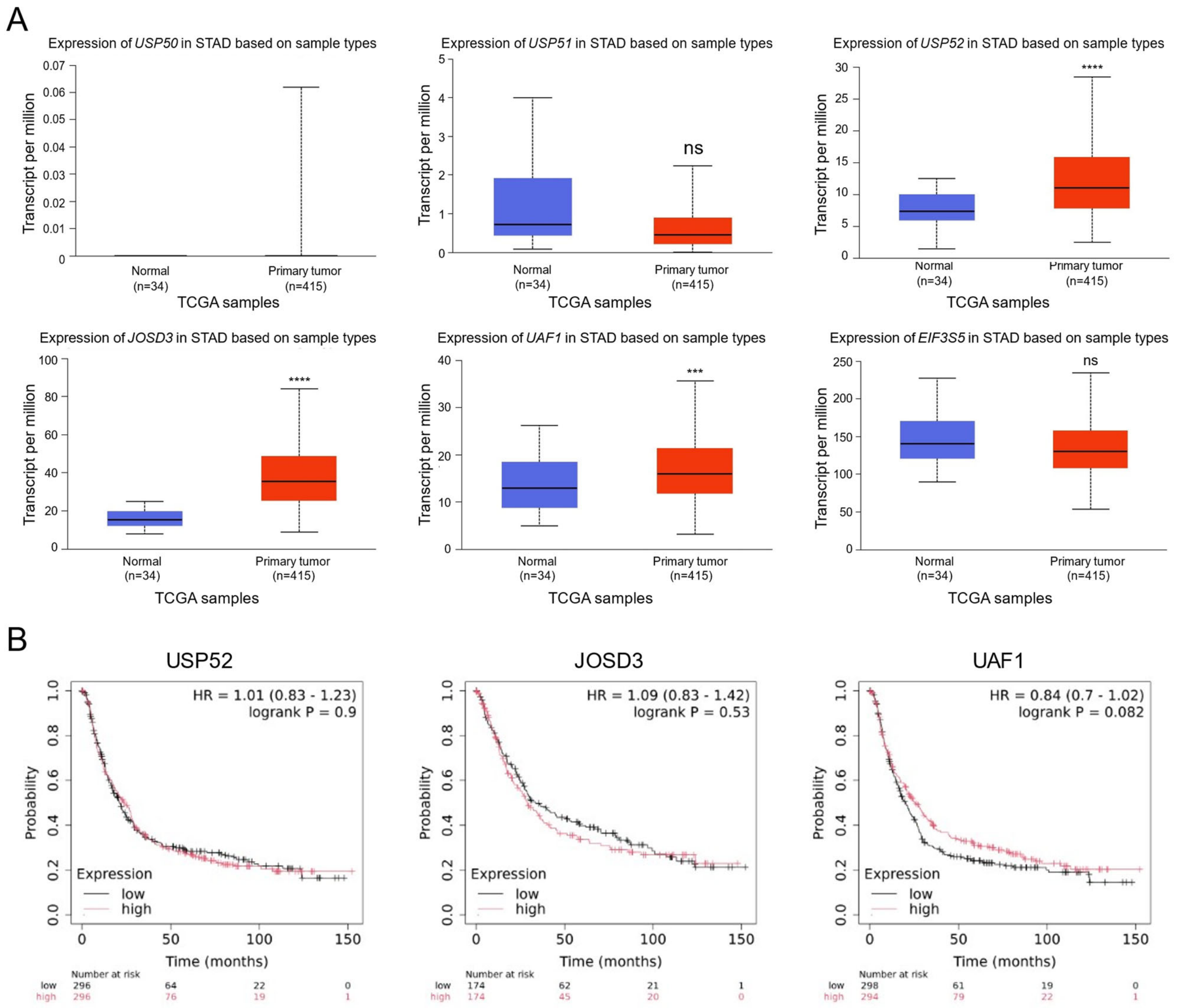

Figure S5. Bioinformatic analysis of the mRNA expression of DUBs, which upregulate RBM39 in the biochemical screen, in gastric cancer tissues and their relationship with the survival of gastric cancer patients.

(A) The mRNA expression of *USP50*, *USP51*, *USP52*, *JOSD3*, *UAF1*, and *EIF3S5* in normal and gastric cancer tissues were obtained from the UALCAN database. *USP52*, *JOSD3*, and *UAF1* mRNAs were highly expressed in gastric cancer tissues. (B) Kaplan-Meier analysis of the relationship between *USP52*, *JOSD3*, or *UAF1* mRNA expression and gastric cancer patient survival. *USP52*, *JOSD3*, and *UAF1* mRNA expression did not affect the survival of gastric cancer patients.

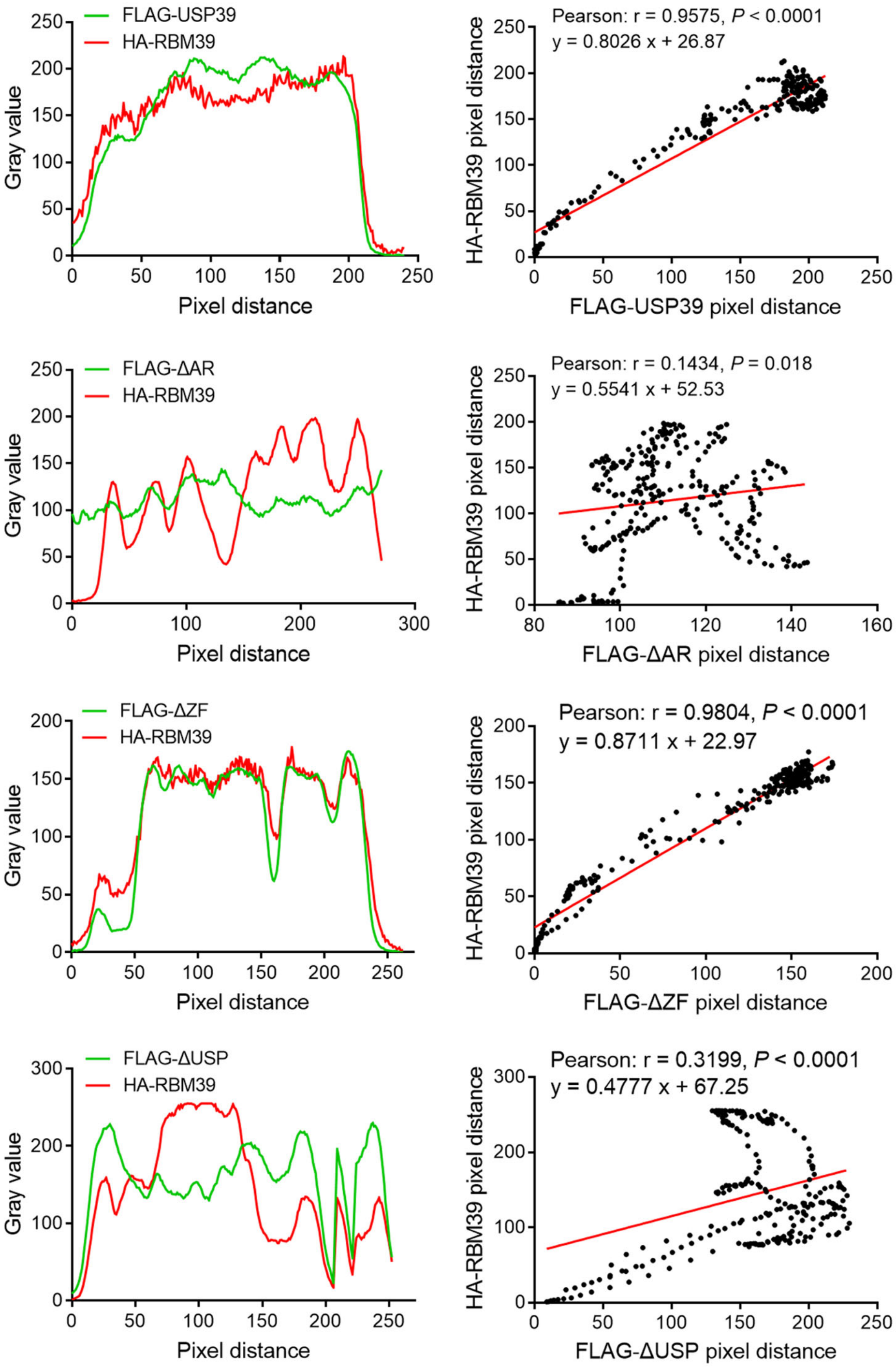

Figure S6. Colocalization analyses for RBM39 and USP39 or its domain deletion mutants.

The colocalization between RBM39 and USP39 or its domain deletion mutants was analyzed using the plot profile tool in ImageJ. Intensity traces from the original images (Figure 5E) were plotted on the left side and their correlation was depicted on the right side. Colocalization coefficients were obtained by Pearson's correlation analysis.

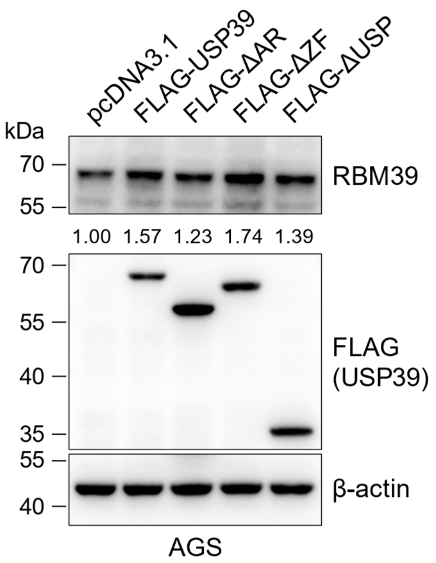

Figure S7. The WT USP39 and RBM39-interacting USP39 mutant (ΔZF) increase RBM39 protein level.

AGS cells were transfected with the indicated plasmids for 48 h and lysed. The cell lysates were immunoblotted with the indicated antibodies. The relative intensity of RBM39 was indicated below the image.

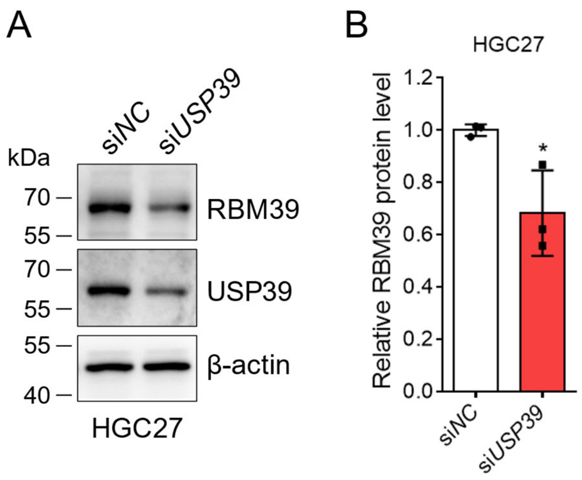

Figure S8. USP39 depletion reduces the RBM39 protein level in HGC27 cells.

(A-B) HGC27 cells transfected with si*NC* or si*USP39* for 48 h and the cell lysates were immunoblotted with the indicated antibodies. Mean ± SD (n = 3), Student's *t*-test with group comparisons, *: $P < 0.05$.

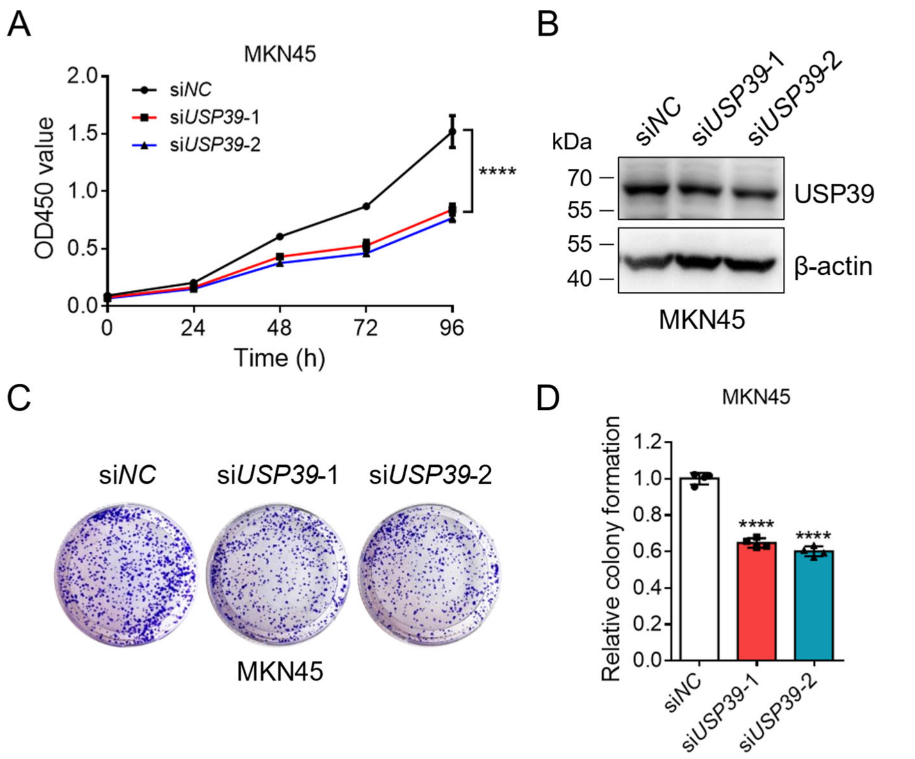

Figure S9. USP39 depletion inhibits the proliferation and colony formation of MKN45 cells.

(A-B) MKN45 cells were transfected with si*NC* or si*USP39* for 24 h and the cell viability was measured with CCK-8. (C-D) 2×10^3 of MKN45 cells in (A) were inoculated in a 35-mm petri dish for colony formation assay. Mean ± SD (n = 4), Two-way ANOVA with Dunnett's multiple comparisons test (A), One-way ANOVA with Dunnett's multiple comparisons test (D), ****: $P < 0.0001$.

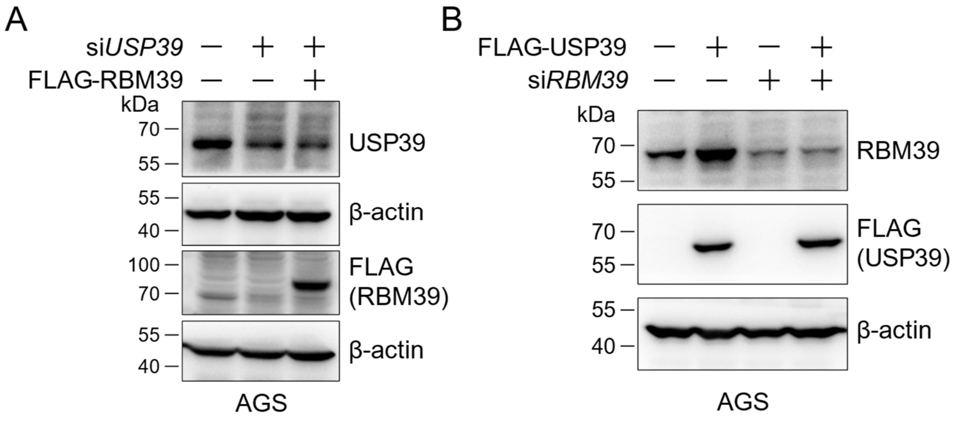

Figure S10. Western blotting analysis of USP39 and RBM39 proteins after transfection of the indicated si*RNA* and plasmid.

(A-B) AGS cells were transfected with the indicated si*RNA* or plasmid for 48 h and the resulting cell lysates were immunoblotted.

Supporting Tables

Table S2. Reagents and antibodies used in this work.

Reagents and antibodies	Cat. #	Company
Ammonium chloride (NH_4Cl)	HY-Y1269	MedChemExpress, USA
Bafilomycin A1 (Baf A1)	HY-100558	MedChemExpress, USA
Bortezomib	HY-10227	MedChemExpress, USA
Cell Counting Kit-8 (CCK-8)	FD3788	Fdbio Science
Chloroquine (CQ)	C6628	Sigma-Aldrich
Cycloheximide (CHX)	HY-12320	MedChemExpress, USA
MG132	HY-13259	MedChemExpress, USA
Alexa Fluor 488 donkey anti-rabbit IgG (H+L)	A21206	Thermo Fisher Scientific, USA
Alexa Fluor 594 goat anti-mouse IgG (H+L)	A11005	Thermo Fisher Scientific, USA
Anti-rabbit IgG	A7016	Beyotime, China
DAPI	D9542	Sigma-Aldrich, USA
FLAG monoclonal antibody	20543-1-AP	Proteintech, USA
FLAG polyclonal antibody	M185-3L	MBL, Japan
GAPDH monoclonal antibody	60004-1-Ig	Proteintech, USA
HA monoclonal antibody	M180-3	MBL, Japan
HA polyclonal antibody	561-5	MBL, Japan
Horseradish peroxidase affiniPure goat anti-rabbit IgG (H+L)	111-035-045	Jackson ImmunoResearch, USA
Horseradish peroxidase affiniPure goat anti-mouse IgG (H+L)	115-035-062	Jackson ImmunoResearch, USA
Myc polyclonal antibody	16286-1-AP	Proteintech, USA
RBM39 polyclonal antibody	21339-1-AP	Proteintech, USA
USP39 polyclonal antibody	23865-1-AP	Proteintech, USA
β-actin monoclonal antibody	66009-1-Ig	Proteintech, USA

Table S3. siRNA used for knockdown.

siRNA	Sequence (5'-3')
si*USP39*-1	sense: UCAAGAGAUUCACUAAGAA
	antisense: UUCUUAGUGAAUCUCUUGA
si*USP39*-2	sense: GCAGUUGUACUUUGCAGUA
	antisense: UACUGCAAAGUACAACUGC
si*RBM39*	sense: GACAGAAAUUCAAGACGUU
	antisense: AACGUCUUGAAUUUCUGUC

Table S4. Primers used for qPCR.

Primer	Sequence (5'-3')
RBM39-Forward	GTCGATGTTAGCTCAGTGCCTC
RBM39-Reverse	ACGAAGCATATCTTCAGTTATG
GAPDH-Forward	GAGTCAACGGATTTGGTCGT
GAPDH-Reverse	TTGATTTTGGAGGGATCTCG
